# Supplementary material for: The role of comorbidities in the associations between air pollution and Alzheimer’s disease: A national cohort study in the American Medicare population
Source: PLoS Med. 2026 Feb 17;23(2):e1004912. doi: 10.1371/journal.pmed.1004912 (PMC12912588; doi:10.1371/journal.pmed.1004912)
Supplement: S1 Table — (DOCX) [file pmed.1004912.s002.docx]

| **S1 Table. ICD-codes for Comorbidities used in CCW database.** | | | |
| --- | --- | --- | --- |
|  | Hypertension | Stroke | Depression |
| ICD-9^1^ | 362.11, 401.0, 401.1, 401.9, 402.00, 402.01, 402.10, 402.11, 402.90, 402.91, 403.00, 403.01, 403.10, 403.11, 403.90, 403.91, 404.00, 404.01, 404.02, 404.03, 404.10, 404.11, 404.12, 404.13, 404.90, 404.91, 404.92, 404.93, 405.01, 405.09, 405.11, 405.19, 405.91, 405.99, 437.2 (any DX on the claim) | 430, 431, 433.01, 433.11, 433.21, 433.31, 433.81, 433.91, 434.00, 434.01, 434.10, 434.11, 434.90, 434.91, 435.0, 435.1, 435.3, 435.8, 435.9, 436, 997.02 (any DX on the claim) | 296.20, 296.21, 296.22, 296.23, 296.24, 296.25, 296.26, 296.30, 296.31, 296.32, 296.33, 296.34, 296.35, 296.36, 296.51, 296.52, 296.53, 296.54, 296.55, 296.56, 296.60, 296.61, 296.62, 296.63, 296.64, 296.65, 296.66, 296.89, 298.0, 300.4, 309.1, 311  (any DX on the claim) |
| ICD-10^1^ | H35.031, H35.032, H35.033, H35.039, I10, I11.0, I11.9, I12.0, I12.9, I13.0, I13.10, I13.11, I13.2, I15.0, I15.1, I15.2, I15.8, I15.9, I67.4, N26.2 (any DX on the claim) | G45.0, G45.1, G45.2, G45.8, G45.9, G46.0, G46.1, G46.2, G46.3, G46.4, G46.5, G46.6, G46.7, G46.8, G97.31, G97.32, I60.00, I60.01, I60.02, I60.10, I60.11, I60.12, I60.20, I60.21, I60.22, I60.30, I60.31, I60.32, I60.4, I60.50, I60.51, I60.52, I60.6, I60.7, I60.8, I60.9, I61.0, I61.1, I61.2, I61.3, I61.4, I61.5, I61.6, I61.8, I61.9, I63.00, I63.011, I63.012, I63.013, I63.019, I63.02, I63.031, I63.032, I63.039, I63.09, I63.10, I63.111, I63.112, I63.113, I63.119, I63.12, I63.131, I63.132, I63.133, I63.139, I63.19, I63.20, I63.211, I63.212, I63.213, I63.219, I63.22, I63.231, I63.232, I63.233, I63.239, I63.29, I63.30, I63.311, I63.312, I63.313, I63.319, I63.321, I63.322, I63.323, I63.329, I63.331, I63.332, I63.333, I63.339, I63.341, I63.342, I63.343, I63.349, I63.39, I63.40, I63.411, I63.412, I63.413, I63.419, I63.421, I63.422, I63.423, I63.429, I63.431, I63.432, I63.433, I63.439, I63.441, I63.442, I63.443, I63.449, I63.49, I63.50, I63.511, I63.512, I63.513, I63.519, I63.521, I63.522, I63.523, I63.529, I63.531, I63.532, I63.533, I63.539, I63.541, I63.542, I63.543, I63.549, I63.59, I63.6, I63.8, I63.81, I63.89, I63.9, I66.01, I66.02, I66.03, I66.09, I66.11, I66.12, I66.13, I66.19, I66.21, I66.22, I66.23, I66.29, I66.3, I66.8, I66.9, I67.841, I67.848, I67.89, I97.810, I97.811, I97.820, I97.821 (any DX on the claim) | F31.30, F31.31, F31.32, F31.4, F31.5, F31.60, F31.61, F31.62, F31.63, F31.64, F31.75, F31.76, F31.77, F31.78, F31.81, F32.0, F32.1, F32.2, F32.3, F32.4, F32.5, F32.9, F33.0, F33.1, F33.2, F33.3, F33.40, F33.41, F33.42, F33.8, F33.9, F34.1, F43.21, F43.23 (any DX on the claim) |
| Number/Type of Claims to Qualify^2^ | At least 1 inpatient/SNF/HHA claim OR 2 HOP/Carrier claims with DX codes | At least 1 inpatient claim OR 2 HOP/Carrier claims with DX codes | At least 1 inpatient, SNF, HHA, HOP, or Carrier claim with DX codes |
| Abbreviations: ICD, International Classification of Diseases; CCW, Chronic Conditions Warehouse. | | | |
| ^1^ICD-10 codes are effective 10/2015; effective dates for ICD-9 codes vary, but are valid through 09/2015. Researchers may be interested in confirming the code(s) of interest in the accompanying claims data files. | | | |
| ^2^SNF refers to skilled nursing facility; HHA refers to home health agency; HOP refers to hospital outpatient. Carrier claims refer to claim types 71 and 72 (not durable medical equipment [DME] claim types 81 or 82), and excludes any claims for which line item Berenson-Eggers Type of Service (BETOS) code variable equals D1A, D1B, D1C, D1D, D1E, D1F, D1G (which is DME), or O1A (which is ambulance services). The intent of the algorithm is to exclude claims where the services do not require a licensed health care professional. When two claims are required, they must occur at least one day apart. DX denotes diagnosis. | | | |
