# Supplementary material for: The role of comorbidities in the associations between air pollution and Alzheimer’s disease: A national cohort study in the American Medicare population
Source: PLoS Med. 2026 Feb 17;23(2):e1004912. doi: 10.1371/journal.pmed.1004912 (PMC12912588; doi:10.1371/journal.pmed.1004912)
Supplement: S2 Table — (DOCX) [file pmed.1004912.s003.docx]

| **S2 Table. Association of PM_2.5_ and confounders with incident AD in the complete model.** | | | |
| --- | --- | --- | --- |
| Variable | Coefficient | Robust se | p-value |
| PM_2.5_ | 0.021 | 0.001 | < 0.001 |
| Year (Ref. 2005) |  |  |  |
| 2006 | -0.100 | 0.014 | < 0.001 |
| 2007 | -0.132 | 0.013 | < 0.001 |
| 2008 | -0.162 | 0.013 | < 0.001 |
| 2009 | -0.204 | 0.013 | < 0.001 |
| 2010 | -0.274 | 0.013 | < 0.001 |
| 2011 | -0.355 | 0.013 | < 0.001 |
| 2012 | -0.388 | 0.013 | < 0.001 |
| 2013 | -0.486 | 0.013 | < 0.001 |
| 2014 | -0.584 | 0.013 | < 0.001 |
| 2015 | -0.578 | 0.013 | < 0.001 |
| 2016 | -0.690 | 0.013 | < 0.001 |
| 2017 | -0.907 | 0.014 | < 0.001 |
| 2018 | -1.261 | 0.014 | < 0.001 |
| Regions [Ref. Midwest] |  |  |  |
| Northeast | 0.005 | 0.007 | 0.457 |
| Southeast | 0.117 | 0.005 | < 0.001 |
| Southwest | 0.173 | 0.007 | < 0.001 |
| West | -0.062 | 0.007 | < 0.001 |
| Hypertension [Ref. 0] | 0.171 | 0.002 | < 0.001 |
| Stroke [Ref. 0] | 0.398 | 0.002 | < 0.001 |
| Depression [Ref. 0] | 0.776 | 0.002 | < 0.001 |
| Smoke rate, % | 0.065 | 0.022 | 0.004 |
| Mean body mass index, kg/m^2^ | 0.012 | 0.002 | < 0.001 |
| Number of hospitals, n | < 0.001 | < 0.001 | 0.402 |
| Population density, people per km^2^ | < 0.001 | < 0.001 | 0.134 |
| Black, % | 0.161 | 0.014 | < 0.001 |
| Median household income, $1000 | < 0.001 | < 0.001 | < 0.001 |
| Renting house or apartment, % | 0.017 | 0.020 | 0.390 |
| Below poverty level, % | -0.045 | 0.050 | 0.373 |
| Not graduated from high school, % | 0.372 | 0.033 | < 0.001 |
| Note: PM_2.5_, fine particulate matter. | | | |
