# Supplementary material for: The role of comorbidities in the associations between air pollution and Alzheimer’s disease: A national cohort study in the American Medicare population
Source: PLoS Med. 2026 Feb 17;23(2):e1004912. doi: 10.1371/journal.pmed.1004912 (PMC12912588; doi:10.1371/journal.pmed.1004912)
Supplement: S3 Table — (DOCX) [file pmed.1004912.s004.docx]

| **S3 Table**. **Subgroup analysis by comorbidities of hazard ratios and 95% CIs of per IQR increase in PM_2.5_ associated with AD in three different cohorts.** | | |
| --- | --- | --- |
|  | HR (95% CI) | P-value for interaction^a^ |
| **PM_2.5_→hypertension→AD cohort** |  |  |
| Overall population | 1.089 (1.082, 1.096) | - |
| Without hypertension | 1.099 (1.089, 1.109) | 0.828 |
| With hypertension | 1.098 (1.090, 1.106) |  |
| **PM_2.5_→stroke→AD cohort** |  |  |
| Overall population | 1.086 (1.079, 1.093) | - |
| Without Stroke | 1.088 (1.082, 1.095) | 0.033 |
| With stroke | 1.102 (1.091, 1.112) |  |
| **PM_2.5_→depression→AD cohort** |  |  |
| Overall population | 1.093 (1.086, 1.100) | - |
| Without depression | 1.092 (1.085, 1.098) | 0.578 |
| With depression | 1.088 (1.079, 1.098) |  |
| Abbreviations: AD, Alzheimer’s disease; CI, confidence interval; HR, hazard ratios; IQR: interquartile range; PM_2.5_, fine particulate matter. | | |
| ^a^P-value for interaction term was estimated by the Wald test. | | |
| Three distinct cohorts were created, ensuring that conditions such as hypertension, stroke, and depression developed after the first year of entry into Medicare and before the onset of AD. These three cohorts align with the cohort used in the mediation analysis. | | |
| Exposure was estimated as mean exposure in the prior 5-year window. | | |
