# Supplementary material for: The role of comorbidities in the associations between air pollution and Alzheimer’s disease: A national cohort study in the American Medicare population
Source: PLoS Med. 2026 Feb 17;23(2):e1004912. doi: 10.1371/journal.pmed.1004912 (PMC12912588; doi:10.1371/journal.pmed.1004912)
Supplement: S4 Table — (DOCX) [file pmed.1004912.s005.docx]

| **S4 Table.** **Subgroup analysis by comorbidities of hazard ratios and 95% CIs of per IQR increase in PM_2.5_ associated with AD, using a restricted outcome definition based on direct AD diagnoses only.** | | |
| --- | --- | --- |
|  | HR (95% CI) | P-value for interaction^b^ |
| **Overall population^a^** | 1.095 (1.089, 1.102) | - |
| Without hypertension | 1.106 (1.096, 1.116) | 0.481 |
| With hypertension | 1.111 (1.103, 1.118) |  |
| Without Stroke | 1.099 (1.092, 1.106) | 0.002 |
| With stroke | 1.117 (1.108, 1.126) |  |
| Without depression | 1.106 (1.099, 1.113) | 0.988 |
| With depression | 1.106 (1.098, 1.114) |  |
| Abbreviations: AD, Alzheimer’s disease; CI, confidence interval; HR, hazard ratios; IQR: interquartile range; PM_2.5_, fine particulate matter. | | |
| ^a^Model was conducted for the overall population. | | |
| ^b^P-value for interaction term was estimated by the Wald test. | | |
| Exposure was estimated as mean exposure in the prior 5-year window. | | |
| All those three comorbidities occur before or in the same year as the diagnosis of AD (e.g., the 'no hypertension' group composed of those who never had hypertension prior to their first diagnosis of AD). | | |
