# Supplementary material for: The role of comorbidities in the associations between air pollution and Alzheimer’s disease: A national cohort study in the American Medicare population
Source: PLoS Med. 2026 Feb 17;23(2):e1004912. doi: 10.1371/journal.pmed.1004912 (PMC12912588; doi:10.1371/journal.pmed.1004912)
Supplement: S5 Table — (DOCX) [file pmed.1004912.s006.docx]

| **S5 Table.** **Two-way decomposition of the association of per 1 µg/m^3^ increase in PM_2.5_ with incident AD by comorbidities using causal mediation analysis under a restricted outcome definition based on direct AD diagnoses only.** | | | |
| --- | --- | --- | --- |
|  | Hypertension | Stroke | Depression |
| Direct effect | 1.017 (1.016, 1.017) | 1.019 (1.018, 1.019) | 1.019 (1.018, 1.019) |
| Indirect effect | 1.0004 (1.0003, 1.0004) | 1.001 (1.001, 1.001) | 1.001 (1.001, 1.001) |
| Total effect | 1.017 (1.017, 1.018) | 1.020 (1.020, 1.021) | 1.019 (1.019, 1.020) |
| Proportion Mediated, % | 2.3 | 6.7 | 3.1 |
| Abbreviations: AD, Alzheimer’s disease; PM_2.5_, fine particulate matter. | | | |
| In this cohort, to ensure that the exposure to mediators contributed to the development of AD, we the first year of cohort entry as the exposure year, followed by a 5-year "clean period" without an AD diagnosis. We restricted to our analysis to the study population who did not move during the follow-up period since the exposure is not time-varying. | | | |
