# Supplementary material for: The role of comorbidities in the associations between air pollution and Alzheimer’s disease: A national cohort study in the American Medicare population
Source: PLoS Med. 2026 Feb 17;23(2):e1004912. doi: 10.1371/journal.pmed.1004912 (PMC12912588; doi:10.1371/journal.pmed.1004912)
Supplement: Attachment — Submitted filename: REVISEDAUG272025PMEDICINE-D-25-02362_R1_reviewer.docx [file pmed.1004912.s007.docx]

PLOS Medicine

# The Role of Comorbidities in the Associations between Air Pollution and Alzheimer's Disease: A National Cohort Study in the American Medicare Population

## 16 Abstract

17 **Background:** Air pollution and several common comorbidities—such as hypertension, stroke,
18 and depression—are established risk factors for Alzheimer’s disease (AD). However, whether
19 these comorbidities mediate or amplify the effects of fine particulate matter (PM2.5) on AD
20 remains unclear. We aimed to investigate whether these conditions modify or mediate the
21 association between long-term PM2.5 exposure and incident AD.
22 **Methods and findings:** We conducted a nationwide cohort including 27.8 million US
23 Medicare beneficiaries aged 65 years and older from 2000 to 2018. Long-term exposure to
24 PM2.5 was assessed using high-resolution air pollution datasets. Cox proportional hazards
25 models were applied to estimate the associations between long-term exposure to PM2.5, incident
26 AD, and comorbidities. The potential for comorbidities to modify and mediate the association
27 between PM2.5 and AD was evaluated by stratified analyses and mediation analysis. We
28 identified approximately 3.0 million incident AD cases. Long-term PM2.5 exposure (5-year
29 moving averages) was associated with increased risk of AD in the overall population [hazard
30 ratio [HR] per interquartile range [IQR, 3.8 µg/m³] increase: 1.085 (95% CI: 1.078, 1.091].
31 This association was slightly stronger in individuals with stroke (HR per IQR increase: 1.105;
32 95% CI: 1.096, 1.114), but there was little effect modification for hypertension and depression.
33 PM2.5 exposure was also significantly associated with higher risks of hypertension, depression,
34 and stroke, all of which were also linked to increased AD risk. However, mediation effects
35 were minimal, with 1.6% of the association between PM₂.₅ and incident AD mediated by
36 hypertension, 4.2% by stroke, and 2.1% by depression.
37 **Conclusions:** Our findings suggest that long-term PM2.5 exposure was associated with
38 increased AD risk, primarily through direct rather than comorbidity-mediated pathways. Stroke
39 may modestly increase susceptibility. These findings highlight the need for air quality
40 interventions as part of dementia prevention strategies in aging populations, especially those
41 facing overlapping environmental and clinical vulnerabilities.
42 **REVIEWER COMMENT**
43 **I fully agreed with your conclusions (please see specific comments later), however I would strongly encourage you to specify the 5y moving average exposure data. In a population >65y,the 5y of pollutant data mostly represent late exposures as 5 y data is not representative of the lifelong exposures and certainly does not include major emission exposures well known to increased AD risk, i.e.occupations, proximity to high traffic roads, wild fires, etc.,**
44 Author summary
45 **Why was this study done?**
46 ⚫	Alzheimer’s disease (AD) is the most common form of dementia and a growing public
47 health challenge, especially in aging populations.
48 ⚫	Long-term exposure to air pollution, particularly fine particulate matter (PM2.5), has been
49 linked to an increased risk of AD, but we don’t fully understand how this risk arises.
50 **REVIEWER COMMENT**
51 Yes, we have several potential pathways to explain how PM2.5 impact the brain, so please modify your statement.
52 **but the issue is complex**
53 ⚫	Common chronic health conditions—such as hypertension, stroke, and depression—are
54 also linked to AD, and may help explain or amplify the effects of air pollution on brain
55 health.
56 ⚫	We aimed to determine whether these conditions act as mediators or modifiers in the
57 relationship between PM2.5 exposure and AD risk.

## 58 What did the researchers do and find?

59 ⚫	We conducted a national cohort study to jointly evaluate both mediation and effect
60 modification by hypertension, stroke, and depression in the association between long-term
61 PM2.5 exposure and incident AD over 27.8 million US Medicare beneficiaries aged 65
62 years and older from 2000 to 2018.
63 ⚫	We found that 5 year exposure to PM2.5 was associated with an increased risk of AD.
64 **REVIEWER COMMENT: Please add that your work is based on 5y data**
65 ⚫	This association was slightly stronger in individuals who had experienced a stroke,
66 suggesting they may be more vulnerable.
67 **REVIEWER COMMENT. Or that indeed PM2.5 impacted directly the stroke event**
68 ⚫	Although all three comorbidities including hypertension, stroke, and depression were
69 associated with both PM2.5 exposure and AD risk, they accounted for only a small
70 proportion of the observed association.
71 **REVIEWER COMMENT:**

**No surprises here, for populations exposed a lifetime to PM2.5 and consequently UFMM and NPs, AD starts very early, at a time where no comorbidities are present and indeed several decades (6,7) before the MDs make the diagnosis.**

## 72 What do these findings mean?

73 ⚫	Air pollution may contribute to AD mostly through direct pathways rather than through
74 other chronic health conditions **and at much earlier ages**
75 ⚫	Individuals with a history of stroke may be especially susceptible to the harmful effects of
76 air pollution on brain health. **YES, indeed**
77 ⚫	Improving air quality could be an important way to prevent dementia and protect older
78 adults—particularly those with existing health risks.
79 **Absolutely**

72

73

## 74 1. Introduction

75 Alzheimer's disease (AD), characterized by a gradual decline in memory, cognition, and
76 behavior, is a progressive neurodegenerative disorder and the most common type of dementia
77 [1]. In 2019, over 5 million people in the United States (US) and approximately 57 million
78 worldwide were affected by AD and related dementias (ADRD) [2]. This number is expected
79 to double in the U.S. and triple globally by 2050 [2]. As there is currently no disease-
80 modifying treatments for ADRD, identifying modifiable risk and protective factors is a
81 critical priority for both clinical practice and public health [3].
82 Emerging evidence indicates that exposure to air pollution—particularly fine particulate
83 matter (PM2.5)—is a novel modifiable risk factor associated with the development of AD and
84 dementia [4-6].
85 **REVIEWER COMMENT**
86 **Key to your findings is the extensive literature linking PM2.5, UFPM and industrial NPs to the pediatric biological development of AD, PD and TDP-43 pathology and key to your study the neurodegeneration overlap is very similar to the one seen in US elderly individuals, a situation you should comment in your paper. In your 27.8 million subjects, I can assure you there are many more AD cases than the 3m incident AD you reported. One simple explanation: AD cases are diagnosed late. Patients come to MD with comorbidities, nobody pays attention to their cognition.**
87 **JAD 2021;82:647-659**
88 **JAD 2024;98:1277-1282**
89 **Environ Res 2018;164;475-487**
90 **Environ Res 2025; 122558**
93 Although the exact mechanisms linking PM2.5 to ADRD are not fully
94 understood, research suggests that neuroinflammation, oxidative stress, and chronic
95 comorbidities may play significant roles in this relationship [7-9].
96 **REVIEWER COMMENT**
97 **I strongly suggest you add a couple of good references addressing PM as a key factor for the development of neurodegenerative common diseases. Yes, we do not fully understand, but there are plenty of references on the subject.**
98 Long-term exposure to
99 PM2.5 has also been shown to increase the risk of chronic diseases such as cardiovascular
100 conditions [10, 11], hypertension [12], and mental health disorders [13, 14]. Furthermore,
101 growing evidence, including our previous analysis of the same Medicare data, suggests that
102 comorbidities—particularly hypertension, stroke, and depression—are strongly associated
103 with higher rates of AD and ADRD [15, 16]. It is plausible to hypothesize that certain
104 comorbidities may contribute to the association of PM2.5 with AD and ADRD.
105 Identifying modifiable intermediates in causal pathways is crucial for public health
106 research, as they present key opportunities for effective population-level interventions. To
107 date, few studies have investigated the role of comorbidities as intermediaries between PM2.5
108 and dementia incidence, resulting in inconsistent results [17-20]. Some studies suggest that
109 cardiovascular diseases (CVD), especially stroke, mediated this relationship [17, 18], while
110 others find no evidence of mediation through hypertension or stroke [19, 20]. However, these
111 studies are constrained by small sample sizes and lack adequate mediation analyses that
112 account for interactions between exposure and potential mediators. Additionally, existing
113 research *has predominantly focused on dementia as a composite outcome rather than AD*
114 *specifically.*
115 **REVIEWER COMMENT**
116 **You have a serious potential problem with your statement: AD is indeed a diagnosis of exclusion on everyday clinical life and unless you tell me all your 27.8m people underwent the current research protocols to diagnose AD, you will have to reword your statement. Moreover, there is a strong overlap with other neurodegenerative disease (JAD2021;82:647-659) thus, please reword your “AD specifically”, please.**
112 **2.1. Study design and population**
113 To better capture the incidence of AD rather than its prevalence, we implemented a
114 “clean period” of five calendar years prior to the diagnosis, consistent with methods from
115 earlier research [16, 21]. Consequently, participants entered the cohort on January 1 of the
116 year following this clean period and were followed until their first AD diagnosis, death, or the
117 end of the follow-up period. The 5-year clean period was excluded from the follow-up time,
118 as participants were not considered at risk for AD during this duration.

**REVIEWER COMMENT:**

112 **The clean period of 5 calendar year does not mean subjects were free of AD, indeed I can assure they were not by the current biological definition.** *The 5-year clean period was excluded from the follow-up time,*

*as participants were not considered at risk for AD during this duration*. **I disagree with you, clean period does not mean they are not at risk for AD.**

## 113 2.2. Exposure assessment

114 High-resolution daily PM2.5 concentrations (24-hour averages) across the U.S. were
115 obtained at a spatial resolution of 1 km × 1 km using spatiotemporal ensemble models that
116 incorporated various machine learning algorithms, including gradient boosting, neural
117 networks, and random forests. Detailed methods can be found in prior research [22]. In brief,
118 the ensemble model was calibrated with numerous predictors, such as land-use data,
119 meteorological variables, chemical transport model simulations, satellite measurements, and
120 monitoring data from the Environmental Protection Agency (EPA) Air Quality Systems
121 (AQS). This approach yielded a robust PM2.5 prediction model, achieving an average cross-
122 validated coefficient of determination (R²) of 0.89 [22].
123 Daily PM2.5 exposures were aggregated by ZIP code, averaged annually, and linked to
124 each Medicare beneficiary according to their ZIP code of residence and follow-up year. We
125 calculated 5-year moving averages of PM2.5 for each individual, treating these as time-varying long-term exposure windows. For example, a participant entering Medicare in 2000
126 and starting follow-up in 2005 following the 5-year clean period without AD, would have
127 PM2.5 estimates based on average concentrations from 2000 to 2004, for their first follow-up
128 year (2005).

**REVIEWER COMMENT:**

**The major problem here is that you are missing all other exposures, including household and occupational ones.**

## 129 2.3. Outcome assessment

130 In this study, we focused on AD as the outcome. We defined AD based on the date of the
131 first recorded diagnosis using either AD diagnosis codes or dementia diagnosis followed by
132 AD, under the assumption that AD is often undercoded initially or diagnosed progressively
133 [16, 21].

**REVIEWER COMMENT**

**That is indeed a major problem.**

## 134 2.4. Comorbidities assessment

136 We examined three comorbidities—hypertension, stroke, and depression—as potential
137 mediators or modifiers in the relationship between PM2.5 exposure and AD, based on previous
138 findings that link these conditions strongly to higher AD rates [16].

**REVIEWER COMMENT**

**Please tell us why you selected depression as a comorbidity?**

139 To examine the potential mediating role of comorbidities, we created three distinct
140 cohorts, ensuring that conditions such as hypertension, stroke, and depression developed after
141 PM2.5 exposure and before the onset of AD.
142 Comorbidities, if present, were required to
143 develop between the first year of entry and the occurrence of AD, to ensure they were
144 potential mediators.
145 **REVIEWER COMMENT**

**From the clinical point of view, it would be unexpected that a comorbidity will have a great AD impact in one year, with exception of stroke. Stroke will be linked to previous vascular dementia in a % of cases and their neurological impact will depend mostly on the extent of the vascular territory involved and the type of stroke.**

148 Mediation requires both that the exposure is associated
149 with subsequent comorbidities, and that comorbidities are associated with subsequent AD. To
150 assess the possibility of mediation, we first assessed the association between time-varying
151 long-term PM2.5 exposure (5-year moving averages) and comorbidities during the follow-up
152 using Cox proportional hazards models. Next, we tested whether individuals with
153 comorbidities were more likely to develop incident AD, also using Cox proportional hazards
154 models.

**REVIEWER COMMENT**

**A major problem with your assumption, is that the 5y pollutant data is short and equally troublesome you have no other data related to occupational or other emissions exposures,previous residency place, etc.,**

274 cases in the cohort occur during the follow up, many potential participants were excluded. In
275 the stroke cohort, which included 26.5 million individuals and recorded 172.1 million person-
276 years of follow-up, around 2.6 million individuals experienced AD events (9.8%) and 5.2
277 million were diagnosed with stroke (19.7%). The depression cohort, consisting of 25.2
278 million individuals and 164.9 million person-years of follow-up, reported approximately 2.3
279 million AD events (9.3%) and 7.2 million depression events (28.5%) during the follow-up
280 period.
281 Table 2 presents the association between long-term exposure to PM2.5 and AD. In the
282 overall population, a higher hazard ratio (HR) for AD was observed with each IQR increase
283 in the 5-year average concentrations of PM2.5, with an HR of 1.085 (95% CI: 1.078, 1.091),
284 after adjusting for all covariates and three comorbidities. In the stratified analyses by
285 comorbidities to assess effect modification, individuals with stroke experienced a somewhat
286 higher risk of AD linked to per IQR increase in PM2.5 exposure, with HR of 1.105 (95% CI:
287 1.096, 1.114) compared to HRs of 1.088 (95% CI: 1.082, 1.095) for those without stroke (P
288 for interaction =0.004). No significant statistical differences were observed in the effect of
289 PM2.5 on AD between individuals with and without hypertension or depression. These
290 associations remained consistent after excluding participants with a history of comorbidities
291 at baseline or those diagnosed with both comorbidities and AD in the same year (S2 Table).
292 Fig 2 shows that the conditions for mediation were present, as per IQR increase in PM2.5
293 was associated with the three comorbidities, and in turn, the comorbidities were associated
294 with AD. Table 3 presents the results of the causal mediation analysis (using a one-unit
295 change in PM2.5 as the metric, not the IQR). Nearly all the overall associations were
296 dominated by direct associations, with minimal evidence that the effect of PM2.5 on AD was
297 mediated through comorbidities. Specifically, approximately 1.6% of the effect was mediated
298 by hypertension, about 4.2% by stroke, and around 2.1% by depression.

| 299 |  |
| --- | --- |
| 300 | **4. Discussion** |
| 301 | This is the first nationwide cohort study in the US to systematically evaluate the dual |
| 302 | role of multiple comorbidities—as both effect modifiers and potential mediators—in the |
| 303 | relationship between long-term PM2.5 exposure and incident AD.  **REVIEWER COMMMENT**  **Three major problems with your study:**  **1.5y PM2.5 is not enough when you are evaluating AD, that takes decades to evolve and progress**  **2.You have no data on occupational and household exposure and certainly on previous residency expoures, proximity to high traffic, use of public transportation,etc,.**  **3.In Medicare populations the diagnosis of AD is late and once is established as clinicians we know of the overlap and thus, AD diagnosis is seldome crystal clear. Moreover, your clean period is meaningless in the common clinica practice. The patient that was seen because osteoporosis, was able to give her name and address, but nobody ran a cognitive test, so the lack of Dx only means nobody check the cognitive status ( we that see every day, across the US).** |

324 However, our analysis revealed that the proportion of the association between PM2.5 and
325 incident AD mediated by these comorbidities was quite small, with mediated proportions of
326 1.6% for hypertension, 2.1% for depression, and 4.2% for stroke.
327 **REVIEWER COMMENT**
328 **This is indeed the most important result of your study. You have to comment about the papers that address the much earlier development of AD in young populations without any evidence of comorbidities.The key for these populations is their exposures from in utero to high PM2.5 concentrations.**
329 **Biological AD starts in infancy and progresses as the subject is exposed to a complex mixture of pollutants and occupational environments.**
330 **Our problem as MDs is that we are diagnosing AD late,very late.**
332 This study has several key strengths. First, it is the first large-scale national cohort study
333 to simultaneously evaluate the roles of hypertension, stroke, and depression in the association
334 between air pollution and incident AD among US Medicare enrollees.

**REVIEWER COMMENT**

335 **Pollution data is too short**
337 Second, we focused
338 on post-exposure incident cases of hypertension, stroke, and depression as potential
339 mediators, rather than their baseline prevalence, to capture the temporal sequence of
340 exposure, mediators, and AD.
341 **REVIEWER COMMENT**
343 *ensuring that conditions such as hypertension, stroke, and depression developed after*
344 *PM2.5 exposure and before the onset of AD.*
345 **That is your other problem. If I have a patient with HBP, I do treat him and fully expect neither vascular dementia nor a stroke affects the patient. Your expectation of** *hypertension, stroke, and depression developed after PM2.5 exposure and before the onset of AD.* **is absolutely not realistic.**
346 **These people have had PM2.5 exposures, but you do not have the data.On the other hand, people are walking with high TAs and they do not know it.**
349 Despite these strengths, our study has several limitations. First, while the exposure
350 prediction model performs well, there is a potential measurement error **because PM2.5 levels**
351 **were assigned based on ZIP code instead of individual residential addresses**.
352 Third, although we accounted for multiple covariates, these data
353 were only available at the ZIP code level. Consequently, residual or unmeasured individual-
354 level confounders, such as alcohol consumption, smoking history, and physical activity, may
355 bias the observed findings.
356 In conclusion, long-term PM2.5 exposure was associated with increased AD risk in a
357 large national cohort, primarily through direct pathways rather than mediation by common
358 comorbidities.
359 **REVIEWER COMMENT**
360 **This is your absolute beautiful finding, take advantage and expand the results using available literature, you will help to change the current paradigm where you need comorbidities to develop AD. WRONG!**

395
