# Supplementary material for: The role of comorbidities in the associations between air pollution and Alzheimer’s disease: A national cohort study in the American Medicare population
Source: PLoS Med. 2026 Feb 17;23(2):e1004912. doi: 10.1371/journal.pmed.1004912 (PMC12912588; doi:10.1371/journal.pmed.1004912)
Supplement: Attachment — Submitted filename: Response to reviewers.docx [file pmed.1004912.s008.docx]

**Response Letter to the Reviewers’ Comments**

**Manuscript reference number:** **PMEDICINE-D-25-02362R1**

**Title: The Role of Comorbidities in the Associations between Air Pollution and Alzheimer's Disease: A National Cohort Study in the American Medicare Population**

The Authors thank the Reviewers for the feedback that they provided and the Editor for the opportunity to respond to these comments. Detailed responses to each comment are given below. Reviewer/Editor comments are in **bold** *italics*. The author’s responses are in plain text. Modifications to the manuscript are in red. Page and line numbers in the responses correspond to changes in the revised manuscript (highlighted in blue).

******************************************************************************

**Comments from the Editor:**

***The reviewers showed interest in your study, but also raised concerns about potential confounders. Furthermore, there were several requests for clarifications and more detail. After discussing the paper with the editorial team and an academic editor with relevant expertise, I'm pleased to invite you to revise the paper in response to the reviewers' comments. We plan to send the revised paper to some or all of the original reviewers, and we cannot provide any guarantees at this stage regarding publication. In addition to these revisions, you may need to complete some formatting changes, which you will receive in a follow up email. A member of our team will be in touch with a set of requests shortly. If you do not receive a separate email within a few days, please assume that checks have been completed, and no additional changes are required.***

Response: We appreciate the opportunity to revise our manuscript. We have carefully considered all comments and suggestions from the Editor and Reviewers, and have addressed them in the revised version. Detailed responses and corresponding revisions are provided below.

**Comments from the Reviewers:**

**Reviewer #1:**

***"The Role of Comorbidities in the Associations between Air Pollution and Alzheimer's Disease: A National Cohort Study in the American Medicare Population" investigates the effect of known Alzheimer's Disease (AD) comorbidities, in air pollution as quantified by PM_2.5_ Stratified and mediation analyses were performed on a nationwide cohort of some 28 million elderly subjects (with 3 million incident AD cases) from 2000 to 2018, to estimate the mediation effect of comorbidities, on the association between PM_2.5_ and AD. In general, minimal mediation effects were found, suggesting that the association between PM_2.5_ exposure and AD is l******argely through direct pathways.***

***Some issues might be considered:***
***Comment 1: In the Introduction, the lack of studies investigating the role of comorbidities as intermediaries between PM_2.5_ and AD is claimed. However, in general, it appears that the following analysis might not be comprehensive enough to draw meaningful conclusions, as acknowledged in the limitations on the presence of large numbers of potential individual confounders (which would seem to possibly have significantly larger impacts on AD prevalence, than the relatively low impact of PM_2.5_). The value of the study might thus be further justified.***
Response: We agree that individual-level potential confounders may influence the risk of Alzheimer’s disease (AD) but are not captured in administrative Medicare data. Nevertheless, we implemented extensive confounding control to the extent possible within this dataset. We controlled for important individual-level potential confounders including age, sex, race, and Medicaid eligibility to minimize bias. We also adjusted for calendar year and geographic variation to account for secular and spatial trends. Finally, we incorporated ZIP code–level socioeconomic indicators, smoking rates, and body mass index (BMI), and additionally controlled for five geographic regions across the United States. Genetic factors are unlikely to be confounders, as they are not plausibly associated with air pollution exposure. We believe that unmeasured individual-level potential confounders, such as smoking and BMI, are likely reasonably well correlated with these area-level and demographic proxies. Moreover, validation studies have shown that area-based socioeconomic indicators yield results consistent in direction and only slightly attenuated compared with those using individual-level SES data, suggesting that any residual misclassification would likely bias our results slightly toward the null [1-3].

Importantly, despite these limitations, our study leverages a large, nationally representative cohort with detailed temporal and geographic exposure data, enabling robust estimation of population-level associations between air pollution, comorbidities, and AD risk. Given the paucity of evidence on the potential intermediary role of comorbidities in this pathway, we believe our findings provide valuable insights and generate hypotheses for future research using more granular individual-level data. Relevant clarifications have been incorporated into the revised manuscript.

**Revised text in the section of Introduction:**

**Page 7, Lines 116–120:** To address this gap, we conducted a nationwide cohort study of more than 27 million older adults in the U.S. to evaluate the roles of three common comorbidities—hypertension, stroke, and depression—as potential mediators and effect modifiers of the association between PM_2.5_ exposure and incident AD, while controlling for multiple potential confounders.

**Revised text in the section of Discussion:**

**Page 21, Lines 452–465:** Third, although we accounted for multiple covariates, some data were only available at the ZIP code level. Consequently, we were able to adjust for some risk factors, such as smoking and BMI, only at the area level, which may have introduced some residual confounding in the PM_2.5_–AD or PM_2.5_–comorbidity associations, particularly if these unmeasured individual behaviors were correlated with air pollution levels. However, we adjusted for individual-level Medicaid eligibility and race, both important indicators of SES, as well as other key individual-level confounders, i.e., age and sex. Moreover, validation literature has shown that area-based socioeconomic indicators yield results consistent in direction and slightly attenuated compared with those using individual-level SES data, suggesting that any residual misclassification would likely bias our results only slightly toward the null [1-3]. Overall, we believe that our control for a large number of individual- and area-level potential confounders was reasonably thorough, and that confounding was unlikely to markedly distort our findings for either the PM_2.5_/AD associations or the PM_2.5_/mediator associations.

***Comment 2: In Section 2.1, it is stated that the open cohorts used had subjects able to enter and leave at any time from 2000 to 2018. It might be clarified as to whether this might bias the cohorts (e.g. correlation between increased socioeconomic status and decreased Medicare/Medicaid participation).***
Response: The open-cohort structure follows the standard Medicare framework, in which beneficiaries enter upon eligibility and exit at death or loss of continuous fee-for-service coverage. This design is standard in large national Medicare-based epidemiologic studies [4, 5] and does not introduce systematic bias, as each individual contributes person-time only while under observation. Because disease incidence is not consistently recorded in Medicare Advantage plans, we restricted follow-up to consecutive fee-for-service enrollment to ensure complete and consistent outcome capture.

Restriction to fee-for-service beneficiaries could possibly affect generalizability to the overall U.S. population aged 65 years and older, due to decreased Medicare participation over time among those with higher socioeconomic status (SES), in favor of, for example, Medicare Advantage. However, this should not bias our findings within the population we studied. It might also not have much effect on generalizability, even if the associations in our data between PM_2.5_ and AD, and PM_2.5_ and comorbidities, were to differ by SES—given that there remains wide SES heterogeneity within the Medicare Fee-for-Service population. We adjusted for both individual-level (Medicaid dual eligibility) and area-level socioeconomic and behavioral covariates at the ZIP code and county levels. We have added related content in our revised manuscript.

**Revised text in the section of Materials and Methods:**

**Page 11, Lines 217–221:** Analyses were stratified by 1-year categories of age at study entry, race, sex, and Medicaid eligibility, and adjusted for the aforementioned covariates, including calendar year and time-updated socioeconomic indicators, to account for temporal changes in population composition and minimize potential bias related to differences in the Medicare/Medicaid population by SES over time.

***Comment 3: The completeness of the data in the cohort might be clarified. In particular, would a diagnosis of AD necessarily be included in the two databases used, or might such data be missing if the patient sought treatment at private institutions?***
Response: Our cohort was constructed from the Medicare Denominator file and the Chronic Conditions Warehouse (CCW) claims data, which together capture all reimbursed inpatient, outpatient, and professional medical encounters for beneficiaries continuously enrolled in Medicare fee-for-service (Parts A and B), regardless of whether care was delivered at public or private institutions. The validated CCW algorithm identifies AD diagnoses from multiple sources—hospital, outpatient, physician, skilled-nursing, and home-health claims—using ICD-9/10 codes (331.0; G30.x). We have added the related content to the revised manuscript.

**Revised text in the section of Discussion:**

**Page 20, Lines 443–445:** Nevertheless, Medicare inpatient and outpatient claims provide near-complete coverage of medical encounters for fee-for-service beneficiaries aged ≥65 years from both public and private health care providers.

***Comment 4: In Section 2.3, it is stated that AD is defined based on either AD diagnosis codes, or dementia diagnosis followed by AD. It might be clarified as to*** ***the distribution of duration between dementia diagnosis and AD, and whether the strict application of AD diagnosis as outcome data would materially affect the findings.***
Response: We defined AD based on either a direct AD diagnosis or a dementia diagnosis followed by AD, consistent with prior nationwide Medicare research based on the validated Chronic Conditions Warehouse (CCW) algorithm [4, 6, 7]. Among participants who had dementia preceding AD, the median interval between the first dementia and subsequent AD diagnosis was approximately 2 years (interquartile range: 1–4 years). To evaluate potential bias, we have conducted a sensitivity analysis restricting the outcome definition to AD diagnosis codes only, excluding AD-preceded dementia cases. The effect estimates for PM_2.5_ exposure were nearly identical in direction and magnitude (see S4-S5 Tables), indicating that inclusion of dementia-preceded AD did not materially affect our findings. This supports the robustness of our primary outcome definition. We have added related content in our revised manuscript.

**Revised text in the section of Materials and Methods:**

**Pages 9–10, Lines 173–178:** We defined AD based on the date of the first recorded diagnosis in the Medicare data, using either AD diagnosis codes or dementia diagnosis followed by AD, under the assumption that AD may be under-recorded initially or diagnosed late [4, 7]. Among participants with dementia preceding AD, the median duration between the first dementia diagnosis and the subsequent AD diagnosis was approximately 2 years (interquartile range [IQR]: 1–4 years).

**Revised text in the section of Materials and Methods:**

**Page 14, Lines 295–298:** We additionally performed a sensitivity analysis restricting the outcome definition to participants with a direct AD diagnosis only, excluding cases where dementia diagnosis preceded AD. This analysis was conducted to evaluate the robustness of our primary findings to alternative case definitions and potential diagnostic delays.

**Revised text in the section of Results:**

**Page 16, Lines 345–348:** In a sensitivity analysis restricted to participants with a direct AD diagnosis only (excluding dementia-preceded cases), both stratification and mediation results were consistent with the main findings (S4-S5 Tables).

***Comment 5: In Section 2.6, the statistical analysis using stratified Cox proportional hazard models with generalized estimating equations is described. It might be clarified as to whether any assumptions regarding (direction of) causality between the comorbidities and outcome (in particular AD -> comorbidity) were made, regarding the models. It is stated that two-way decomposition causal mediation methods were applied, with the results presented in Table 3. However, this does not appear to show the direction(s) of causality. This might be clarified.***

Response: We agree that causal interpretation requires clear temporal ordering between exposure, mediator, and outcome. In our study, temporal ordering was explicitly specified as PM_2.5_ → comorbidity → AD, consistent with established biological and epidemiological evidence that air pollution contributes to cardiovascular, cerebrovascular, and psychiatric conditions, which in turn increase AD risk [8-10]. To minimize the possibility of reverse causation (AD → comorbidity), we implemented a 5-year clean period before cohort entry, excluding individuals with pre-existing AD or any of the three comorbidities of interest. PM_2.5_ exposure was defined as the exposure in the first year of the clean period, and the cohort was restricted to non-movers. During follow-up, comorbidities were treated as time-varying variables and were required to have occurred prior to AD diagnosis in all mediation analyses, and after PM_2.5_ exposure. This design enforces the temporal sequence required for causal mediation.

The two-way decomposition causal mediation analysis (Table 3) was conducted under this temporal assumption as described above, partitioning the total effect of PM_2.5_ into direct effects (not through comorbidities) and indirect effects (through comorbidities). Because the mediators were measured prior to AD onset, the direction of causality in the models is unidirectional—from exposure to mediator to outcome. We expanded our text in Methods about the temporal sequence.

**Revised text in the section of Materials and Methods:**

**Pages 12–13, Lines 228–265:** To examine the potential mediating role of comorbidities, we created three distinct cohorts, ensuring that conditions such as hypertension, stroke, and depression developed after PM_2.5_ exposure and before the onset of AD. Participants could contribute to more than one cohort if they developed multiple comorbidities, but each comorbidity was modeled independently as a potential mediator. Joint mediation across multiple comorbidities was not evaluated.

To then assess the possibility of mediation, we first assessed the association between time-varying PM_2.5_ exposure (5-year moving averages) and comorbidities during the follow-up using Cox proportional hazards models. Next, we tested whether individuals with comorbidities were more likely to develop incident AD, also using Cox proportional hazards models.

We assumed a temporal causal pathway of PM_2.5_→comorbidity→AD. To minimize the potential for reverse causation (AD→comorbidity), comorbidities were required to occur sometime prior to AD diagnosis. Comorbidities, if present, were required to develop between the first year of entry and the occurrence of AD, to ensure they were potential mediators. This temporal structure was designed to ensure proper temporal ordering rather than to imply short-term causal effects. In practice, these comorbidities were first diagnosed in our mediation analysis several years before the first recorded AD diagnosis in the Medicare data. To ensure that the comorbidities followed exposure, exposure for comorbidity analysis was defined as fixed, and based on the PM_2.5_ level of the participant's zip code at the year of the start of the clean period. Furthermore, participants were required not to have moved out of their original zip code during follow-up (the percentage of non-movers in our cohort was 84.6%), following the method of a previous analysis of mediation [10]. By requiring no movement during follow-up, we increased the likelihood that exposure at entry year was representative of later exposure. Using exposure at the start of the clean period and restricting the cohort to individuals without comorbidities at year of entry helped ensure that comorbidities developed after exposure.

Participants were excluded from the mediation analysis if: 1) they had a history of comorbidities at the start of the clean period. (i.e., at the same time that exposure was defined to ensure that the comorbidity followed exposure) or 2) they were diagnosed with the comorbidity in the same year as their AD diagnosis (as we could not know if the comorbidity preceded the AD, a requirement for possible mediation). These conditions fulfilled the mediation criteria that the exposure is associated with subsequent comorbidities and that comorbidities are associated with subsequent AD.

After assessing the possibility of mediation and finding it to be plausible, we then analyzed the mediation effect using the product method, and applied two-way decomposition causal mediation methods, considering potential interactions between exposure and mediator [11].

***Comment 6: In Section 2.6, it is mentioned that three distinct cohorts were created, one for each comorbidity. It might be clarified as to whether subjects may possess multiple comorbidities, and if so, whether they would be included in multiple cohorts. In that latter case, how is mediation on multiple comorbidities performed?***
Response: Indeed, some participants developed more than one comorbidity (e.g., both hypertension and stroke) during follow-up. As noted in response to Comment 5 above, to isolate the mediating role of each comorbidity, we created three independent analytic cohorts, each restricted to individuals who were free of the specific comorbidity at baseline and followed them until its onset or AD diagnosis. Therefore, a participant could contribute to more than one cohort, but their follow-up time and outcome were analyzed separately in each disease-specific model.

This approach allows assessment of each comorbidity’s mediating effect independently while maintaining consistent exposure and outcome definitions. Because the mediators were evaluated in separate models, joint mediation (i.e., combined or interactive effects of multiple comorbidities) was not estimated. Although there are some new methods for looking at multiple mediators, we did not pursue them here because the observed indirect effects of individual comorbidities were very small, suggesting that additional multiple-mediator modeling would be unlikely to materially change the conclusions while adding model complexity. Our goal was to identify which comorbidity pathways contribute most to the overall PM_2.5_–AD association rather than to quantify their simultaneous effects. We have added this clarification in the revised manuscript.

**Revised text in the section of Materials and Methods:**

**Page 12, Lines 230–233:** Participants could contribute to more than one cohort if they developed multiple comorbidities, but each comorbidity was modeled independently as a potential mediator. Joint mediation across multiple comorbidities was not evaluated.

***Comment 7: In Section 2.6, the assumptions on variables a, a* and c might be explained further.***

Response: In our study, we applied VanderWeele’s two-way decomposition causal mediation framework [11] using two Cox proportional hazards models—one for the exposure–mediator relationship and another for the mediator–outcome relationship. In this framework, a denotes the exposure level of interest, a* denotes the reference exposure level, and c represents the mean values of covariates used for standardization. Accordingly, we set a = 1, a* = 0, and c equal to the covariate means when estimating the controlled direct and indirect effects of PM_2.5_. These assumptions are consistent with standard applications of the two-way decomposition method under Cox modeling [11], where the focus is on the relative effect scale rather than absolute risk. We have clarified this explanation in the revised manuscript.

**Revised text in the section of Materials and Methods:**

**Page 14, Lines 282–285:** In the equation, “a” represents the exposure level of interest, “a*” represents the reference exposure level, and “c” denotes the mean of the covariates used for standardization. Accordingly, we set a = 1, a* = 0, and c equal to the covariate means to estimate the controlled direct and indirect effects under this framework.

***Reviewer #2:***

***Background:*** *Air pollution and several common comorbidities—such as hypertension, stroke, and depression—are established risk factors for Alzheimer’s disease (AD). However, whether these comorbidities mediate or amplify the effects of fine particulate matter (PM_2.5_) on AD remains unclear. We aimed to investigate whether these conditions modify or mediate the association between long-term PM_2.5_ exposure and incident AD.*

***Methods and findings:*** *We conducted a nationwide cohort including 27.8 million US Medicare beneficiaries aged 65 years and older from 2000 to 2018. Long-term exposure to PM_2.5_ was assessed using high-resolution air pollution datasets. Cox proportional hazards models were applied to estimate the associations between long-term exposure to PM_2.5_, incident AD, and comorbidities. The potential for comorbidities to modify and mediate the association between PM_2.5_ and AD was evaluated by stratified analyses and mediation analysis.* *We identified approximately 3.0 million incident AD cases. Long-term PM_2.5_ exposure (5-year moving averages) was associated with increased risk of AD in the overall population [hazard ratio [HR] per interquartile range [IQR, 3.8 µg/m³] increase: 1.085 (95% CI: 1.078, 1.091]. This association was slightly stronger in individuals with stroke (HR per IQR increase: 1.105; 95% CI: 1.096, 1.114), but there was little effect modification for hypertension and depression. PM_2.5_ exposure was also significantly associated with higher risks of hypertension, depression, and stroke, all of which were also linked to increased AD risk. However, mediation effects were minimal, with 1.6% of the association between PM₂.₅ and incident AD mediated by hypertension, 4.2% by stroke, and 2.1% by depression.*

***Conclusions:*** *Our findings suggest that long-term PM_2.5_ exposure was associated with increased AD risk, primarily through direct rather than comorbidity-mediated pathways. Stroke may modestly increase susceptibility. These findings highlight the need for air quality interventions as part of dementia prevention strategies in aging populations, especially those facing overlapping environmental and clinical vulnerabilities.*

***Comment 1: I fully agreed with your conclusions (please see specific comments later), however I would strongly encourage you to specify the 5y moving average exposure data. In a population >65y, the 5y of pollutant data mostly represent late exposures as 5 y data is not representative of the lifelong exposures and certainly does not include major emission exposures well known to increased AD risk, i.e.occupations, proximity to high traffic roads, wild fires, etc.,***
Response: We agree that the 5-year moving average exposure represents late-life rather than lifelong exposure. We have revised the text throughout the manuscript to use more precise wording (e.g., “5-year average exposure” instead of “long-term exposure”) in our revised manuscript. We are also not able to estimate exposures earlier in life for most study subjects, as our ZIP code–level exposure data only extend back to 2000, which we have also noted at the end of Section on exposure assessment.

*Why was this study done?*

- *Alzheimer’s disease (AD) is the most common form of dementia and a growing public health challenge, especially in aging populations.*
- *Long-term exposure to air pollution, particularly fine particulate matter (PM2.5), has been*

*linked to an increased risk of AD, but we don’t fully understand how this risk arises.*
***Comment 2: Yes, we have several potential pathways to explain how PM_2.5_ impact the brain, so please modify your statement. but the issue is complex.***
Response: We agree that several biological pathways may explain how PM_2.5_ affects the brain. We have revised the text to reflect that these mechanisms—such as neuroinflammation, oxidative stress, and vascular injury—are complex and not yet fully understood.

**Revised text in the section of Author summary:**

**Page 4, Lines 47–50:** Exposure to air pollution, particularly fine particulate matter (PM_2.5_), has been linked to an increased risk of AD, likely through pathways such as neuroinflammation, oxidative stress, and vascular injury, though these mechanisms remain complex and not fully understood.

*What did the researchers do and find?*

- *We conducted a national cohort study to jointly evaluate both mediation and effect modification by hypertension, stroke, and depression in the association between long-term PM_2.5_ exposure and incident AD over 27.8 million US Medicare beneficiaries aged 65 years and older from 2000 to 2018.*
- *We found that long-term exposure to PM_2.5_ was associated with an increased risk of AD.*

***Comment 3: Please add that your work is based on 5y data***

Response: We have revised the text accordingly to specify that the exposure was based on 5-year moving average PM_2.5_ data prior to AD occurrence.

**Revised text in the section of Author summary:**

**Page 4, Lines 57–62:**

- We conducted a national cohort study to jointly evaluate both mediation and effect modification by hypertension, stroke, and depression in the association between 5-year time-varying average PM_2.5_ exposure and incident AD over 27.8 million US Medicare beneficiaries aged 65 years and older from 2000 to 2018.
- We found that 5-year average PM_2.5_ exposure prior to AD onset was associated with an increased risk of AD.
- *This association was slightly stronger in individuals who had experienced a stroke, suggesting they may be more vulnerable.*

***Comment 4: Or that indeed PM_2.5_ impacted directly the stroke event***

Response: We agree with the reviewer that PM_2.5_ exposure may directly increase the risk of stroke (see Fig 2), which in turn elevates the risk of AD — this was indeed the focus of our mediation analysis. However, the effect modification we observed, whereby PM_2.5_ shows a stronger association with AD among individuals with a history of stroke compared with those without, represents an independent finding that is separate from the mechanistic pathway evaluated in the mediation analysis.

- *Although all three comorbidities including hypertension, stroke, and depression were associated with both PM_2.5_ exposure and AD risk, they accounted for only a small proportion of the observed association.*

***Comment 5: No surprises here, for populations exposed a lifetime to PM_2.5_ and consequently UFMM and NPs, AD starts very early, at a time where no comorbidities are present and indeed several decades (6,7) before the MDs make the diagnosis.***

Response: We agree with the Reviewer’s comment that PM_2.5_ exposure may contribute to Alzheimer’s disease long before clinical diagnosis. Nonetheless, we found that the 5-year average exposure prior to disease onset was associated with an increased risk. This association could partly reflect the correlation between this recent 5-year exposure window and earlier air pollution levels. We have added text to the Discussion of our revised manuscript.

**Revised text in the section of Discussion:**

**Page 20, Lines 438–441:** Furthermore, we examined 5-year average exposure immediately preceding disease onset and were unable to estimate exposures earlier in life due to the lack of historical exposure data. It is likely that the disease process began earlier, and our findings may therefore reflect the correlation of relatively recent exposure with past exposure levels.

*What do these findings mean?*

- *Air pollution may contribute to AD mostly through direct pathways rather than through other chronic health conditions.*

***Comment 6: and at much earlier ages***

Response: As noted above for Reviewer 2, Comment 5, we agree that the adverse effects of air pollution on Alzheimer’s disease (AD) may begin much earlier in life, long before clinical diagnosis. Our study focused on older adults in the Medicare population, capturing the late-life stage of AD development. Because our dataset lacks information on early-life or midlife exposures, we were unable to directly evaluate these earlier effects; therefore, this aspect was not incorporated into the current analysis. Relevant text has been added to the Discussion, as noted above for Comment 5.

- *Individuals with a history of stroke may be especially susceptible to the harmful effects of air pollution on brain health.*

***Comment 7: YES, indeed***

Response: We are glad the Reviewer agrees here.

- *Improving air quality could be an important way to prevent dementia and protect older adults—particularly those with existing health risks.*

***Comment 8: Absolutely***

Response: We are glad the Reviewer agrees here.

*Emerging evidence indicates that exposure to air pollution—particularly fine particulate matter (PM_2.5_)—is a novel modifiable risk factor associated with the development of AD and dementia* [12-14]*.*

***Comment 9: Key to your findings is the extensive literature linking PM2.5, UFPM and industrial NPs to the pediatric biological development of AD, PD and TDP-43 pathology and key to your study the neurodegeneration overlap is very similar to the one seen in US elderly individuals, a situation you should comment in your paper. In your 27.8 million subjects, I can assure you there are many more AD cases than the 3m incident AD you reported. One simple explanation: AD cases are diagnosed late. Patients come to MD with comorbidities, nobody pays attention to their cognition.***

***JAD 2021;82:647-659***

***JAD 2024;98:1277-1282***

***Environ Res 2018;164;475-487***

***Environ Res 2025; 122558***

Response: That evidence shows that pediatric and young adult populations living in highly polluted environments already exhibit Alzheimer’s disease (AD) related lesions, including amyloid-β (Aβ) plaques, hyperphosphorylated tau tangles, α-synuclein (α-syn) aggregates, and TAR DNA/RNA-binding protein 43 (TDP-43), that closely parallel those observed in late-life neurodegeneration [15-18]. These findings suggest a life-course continuum in which sustained particulate exposures may initiate early neural injury and inflammatory processes that evolve silently for decades before manifesting clinically in older adulthood. Our study captures the late-life stage of this continuum, quantifying how long-term PM_2.5_ exposure during older adulthood relates to clinically diagnosed AD and dementia in the national Medicare population. We agree that many of these “incident” cases likely represent late recognition of long-evolving disease, and we have clarified this in the Introduction and have added the above citations in our revised manuscript.

**Revised text in the section of Introduction:**

**Page 6, Lines 85–95:** Emerging evidence indicates that exposure to air pollution—particularly fine particulate matter (PM_2.5_)—is a novel modifiable risk factor associated with the development of AD and dementia [12-14]. Although the exact mechanisms linking PM_2.5_ to ADRD are not fully understood, research suggests that neuroinflammation, oxidative stress, and chronic comorbidities may play significant roles in this relationship [19-21]. Moreover, neuropathological studies indicate that exposure to PM_2.5_, ultrafine particles, and industrial nanoparticles is associated with early deposition of amyloid-β (Aβ), hyperphosphorylated tau tangles, α-synuclein, and TAR DNA/RNA-binding protein 43 (TDP-43) in children and young adults [15-17]. These pathological features closely resemble those observed in older adults and support a life-course continuum of pollution-related neurodegeneration that may culminate in late-life clinical ADRD [18].

*Although the exact mechanisms linking PM_2.5_ to ADRD are not fully understood, research suggests that neuroinflammation, oxidative stress, and chronic comorbidities may play significant roles in this relationship* [19-21]*.*

***Comment 10: I strongly suggest you add a couple of good references addressing PM as a key factor for the development of neurodegenerative common diseases. Yes, we do not fully understand, but there are plenty of references on the subject.***

Response: As noted above in our response to Reviewer 2, Comment 9, we have added relevant mechanistic descriptions and supporting references in the Introduction to emphasize that exposure to fine particulate matter can contribute to neurodegenerative changes beginning early in life.

*Additionally, existing research has predominantly focused on dementia as a composite outcome rather than AD specifically.*

***Comment 11: You have a serious potential problem with your statement: AD is indeed a diagnosis of exclusion on everyday clinical life and unless you tell me all your 27.8m people underwent the current research protocols to diagnose AD, you will have to reword your statement. Moreover, there is a strong overlap with other neurodegenerative disease (JAD2021;82:647-659) thus, please reword your “AD specifically”, please.***

Response: We agree that AD is largely a clinical diagnosis of exclusion, and that diagnostic overlap with other neurodegenerative dementias (e.g., vascular, Parkinson’s disease, frontotemporal dementias) is common in real-world settings. Our definition of AD was based on the first recorded AD diagnosis in Medicare claims using the validated Chronic Conditions Warehouse (CCW) algorithm, which identifies AD according to ICD-9/10 codes and has been widely used in large-scale population studies. As such, our outcome represents clinically recorded AD rather than pathologically confirmed AD, and we have revised the corresponding sentence to clarify this distinction. We have also cited Calderón-Garcidueñas et al. (2024) [15] to acknowledge the overlapping neuropathology across neurodegenerative diseases.

**Revised text in the section of Introduction:**

**Page 7, Lines 109–113:** Additionally, much existing research has focused on dementia as a composite outcome, whereas our study specifically examined clinically diagnosed AD based on validated Medicare algorithms, although some degree of diagnostic overlap with other neurodegenerative dementias is certainly possible [15].

*To better capture the incidence of AD rather than its prevalence, we implemented a “clean period” of five calendar years prior to the diagnosis, consistent with methods from earlier research* [4, 7]*. Consequently, participants entered the cohort on January 1 of the year following this clean period and were followed until their first AD diagnosis, death, or the end of the follow-up period. The 5-year clean period was excluded from the follow-up time, as participants were not considered at risk for AD during this duration.*

***Comment 12: The clean period of 5 calendar year does not mean subjects were free of AD, indeed I can assure they were not by the current biological definition. The 5-year clean period was excluded from the follow-up time, as participants were not considered at risk for AD during this duration. I disagree with you, clean period does not mean they are not at risk for AD.***

Response: As noted in response to earlier comments, we acknowledge that AD has a long prodromal phase, and many older adults may already be experiencing underlying pathology or cognitive decline before a clinical diagnosis is recorded. Therefore, the 5-year “clean period” in our study does not imply that participants were biologically free of AD or at no risk during this time; it simply indicates that they were free of a Medicare-diagnosed AD record during this period. This approach follows standard practice in claims-based research using the Medicare Chronic Conditions Warehouse (CCW) [4-6, 22]. The clean period is a methodological strategy designed to minimize—though certainly not entirely eliminate—the misclassification of prevalent cases as incident cases. A 5-year exclusion window helps identify the first recorded diagnosis more accurately, consistent with prior large-scale Medicare studies [4-6, 22]. We have revised the Methods section to clarify that the clean period represents a claims-based exclusion window, rather than an indication that participants were free of biological risk.

**Revised text in the section of Materials and methods:**

**Page 8, Lines 143–147:** To better capture newly recorded AD diagnoses rather than previously documented cases, we implemented a 5-year “clean period” prior to cohort entry, consistent with prior Medicare-based research [4, 7]. This approach aims to minimize misclassification of prevalent cases as incident based on Medicare claims, but does not imply that participants were biologically free of early disease during this period.

*Daily PM_2.5_ exposures were aggregated by ZIP code, averaged annually, and linked to each Medicare beneficiary according to their ZIP code of residence and follow-up year. We calculated 5-year moving averages of PM_2.5_ for each individual, treating these as time-varying long-term exposure windows. For example, a participant entering Medicare in 2000 and starting follow-up in 2005 following the 5-year clean period without AD, would have PM_2.5_ estimates based on average concentrations from 2000 to 2004, for their first follow-up year (2005).*

***Comment 13: The major problem here is that you are missing all other exposures, including household and*** ***occupational ones.***

Response: We fully agree that our exposure assessment, which is based on ambient PM_2.5_ concentrations estimated at the residential ZIP-code level, does not capture individual-level household or occupational exposures. This limitation is inherent to large-scale administrative studies such as Medicare, where detailed indoor and occupational exposure data are unavailable. Nevertheless, ambient PM_2.5_ represents the major contributor to total exposure and has been extensively validated against ground monitoring and satellite-based models at high spatial resolution (1 km × 1 km). We have added corresponding text in the Discussion section to acknowledge this limitation and note that any resulting exposure misclassification is likely to be non-differential and would bias the associations toward the null.

**Revised text in the section of** **Discussion:**

**Page 20, Lines 431–438:** Additionally, our exposure assessment captured only outdoor ambient PM_2.5_ concentrations and did not account for household or occupational exposures (e.g., cooking, heating, or workplace sources), which may contribute to total exposure but are unavailable at the national scale. These unmeasured sources could lead to exposure misclassification, although existing data indicate that ambient and total personal exposures are reasonably well correlated (r = 0.60) based on U.S. data [23]. Such misclassification is likely non-differential with respect to AD outcomes and would bias our estimates toward the null.

*In this study, we focused on AD as the outcome. We defined AD based on the date of the first recorded diagnosis using either AD diagnosis codes or dementia diagnosis followed by AD, under the assumption that AD is often undercoded initially or diagnosed progressively* [4, 7]*.*

***Comment 14: That is indeed a major problem.***

Response: As noted above, we agree that defining AD using diagnostic codes in administrative claims data has inherent limitations, as AD is often undercoded or clinically diagnosed only at a later stage. Our study therefore captures clinically recorded AD rather than pathologically confirmed AD, consistent with prior nationwide Medicare research based on the validated Chronic Conditions Warehouse (CCW) algorithm [4, 6, 7]. In response to comments above, we have revised the text to clarify that sub-clinical AD is likely present for a long time prior to clinical diagnosis. We have also clarified in the revised Methods and Discussion that our definition represents the first clinically recorded AD diagnosis, acknowledging that diagnostic delay remains a limitation of all claims-based studies.

Regarding whether the Medicare AD diagnoses themselves are accurate or suffer from poor sensitivity, in our previous work, we conducted two sensitivity analyses to evaluate the potential influence of outcome misclassification—one using a rate-based linear regression model and another adjusting case counts according to Medicare’s reported sensitivity and specificity—and found that the corrections had minimal impact on effect estimates [4]. These results suggest that any misclassification in AD identification would likely bias the observed associations slightly toward the null rather than produce spurious findings. See the revised text below regarding these sensitivity analyses.

**Revised text in the section of Materials and methods:**

**Pages 9–10, Lines 173–178:** In this study, we focused on AD as the outcome. We defined AD based on the date of the first recorded diagnosis in the Medicare data, using either AD diagnosis codes or dementia diagnosis followed by AD, under the assumption that AD may be under-recorded initially or diagnosed late [4, 7]. Among participants with dementia preceding AD, the median duration between the first dementia diagnosis and the subsequent AD diagnosis was approximately 2 years (interquartile range [IQR]: 1–4 years).

**Revised text in the section of Discussion:**

**Pages 20–21, Lines 442–452:** Second, using administrative records to identify disease may lead to outcome misclassification. Nevertheless, Medicare inpatient and outpatient claims provide near-complete coverage of medical encounters for fee-for-service beneficiaries aged ≥65 years from both public and private health care providers. Previous literature has shown that AD diagnoses in Medicare data have high specificity (95%) and moderate sensitivity (64%), supporting their reliability for population-based research [24]. Moreover, in our previous work on air pollution and AD, Shi et al. [4] conducted two sensitivity analyses to address this issue: one using a linear regression model based on rates, and the other using prior estimates of Medicare's sensitivity and specificity to adjust the case counts. They found that correcting for misclassification of outcome did not significantly alter the results, suggesting that such misclassification likely biased our findings slightly toward the null.

*We examined three comorbidities—hypertension, stroke, and depression—as potential mediators or modifiers in the relationship between PM_2.5_ exposure and AD, based on previous findings that link these conditions strongly to higher AD rates* [7]*.*

***Comment 15: Please tell us why you selected depression as a comorbidity?***

Response: We included depression as one of the three comorbidities based primarily on strong epidemiologic evidence linking it to Alzheimer’s disease (AD). A large-scale Medicare study by Steenland et al. (2023) [7] identified depression as one of the comorbidities most strongly associated with AD, showing among the highest hazard ratios across nine chronic conditions examined. Similarly, the 2024 Alzheimer’s Disease Facts and Figures report [25] lists depression as a leading and potentially modifiable risk factor for AD and related dementias. In our dataset, depression was also one of the most prevalent comorbidities (34.9%, see Table 1) in the AD cohort. We have clarified this point in the Methods section of our revised manuscript.

**Revised text in the section of Materials and methods:**

**Page 10, Lines 185–188:** We examined three comorbidities—hypertension, stroke, and depression—as potential mediators or modifiers in the relationship between PM_2.5_ exposure and AD, based on previous findings that link these conditions strongly to higher AD rates [7] and its recognition as a potentially modifiable risk factor for dementia [25].

*To examine the potential mediating role of comorbidities, we created three distinct cohorts, ensuring that conditions such as hypertension, stroke, and depression developed after PM2.5 exposure and before the onset of AD. Comorbidities, if present, were required to develop between the first year of entry and the occurrence of AD, to ensure they were potential mediators.*

***Comment 16: From the clinical point of view, it would be unexpected that a comorbidity will have a great AD impact in one year, with exception of stroke. Stroke will be linked to previous vascular dementia in a % of cases and their neurological impact will depend mostly on the extent of the vascular territory involved and the type of stroke.***

Response: We agree that, biologically, most chronic comorbidities such as hypertension and depression are unlikely to exert substantial effects on AD risk within a very short time frame, except for stroke, which can have more immediate neurological consequences on vascular dementia. There is no assumption in our analysis that comorbidities will precede AD by only one year. Rather, our approach was designed to ensure the correct temporal ordering required for mediation analysis—specifically, that comorbidities occur after exposure to PM₂.₅ but before the onset of AD. This temporal structure allows us to properly assess whether these comorbidities may serve as intermediate conditions through which long-term PM_2.5_ exposure influences AD risk, while minimizing the possibility of reverse causation. In practice, the comorbidities included in our cohorts typically developed several years prior to the first recorded AD diagnosis in the Medicare data. We have clarified this point in the revised Methods section.

**Revised text in the section of Materials and methods:**

**Page 12, Lines 239–245:** We assumed a temporal causal pathway of PM_2.5_→comorbidity→AD. To minimize the potential for reverse causation (AD→comorbidity), comorbidities were required to occur sometime prior to AD diagnosis. Comorbidities, if present, were required to develop between the first year of entry and the occurrence of AD, to ensure they were potential mediators. This temporal structure was designed to ensure proper temporal ordering rather than to imply short-term causal effects. In practice, these comorbidities were first diagnosed in our mediation analysis several years before the first recorded AD diagnosis in the Medicare data.

*Mediation requires both that the exposure is associated with subsequent comorbidities, and that comorbidities are associated with subsequent AD. To assess the possibility of mediation, we first assessed the association between time-varying long-term PM_2.5_ exposure (5-year moving averages) and comorbidities during the follow-up using Cox proportional hazards models. Next, we tested whether individuals with comorbidities were more likely to develop incident AD, also using Cox proportional hazards models.*

***Comment 17: A major problem with your assumption, is that the 5y pollutant data is short and equally troublesome you have no other data related to occupational or other emissions exposures,previous residency place, etc.,***

Response: Please see our earlier responses addressing these points.

*This is the first nationwide cohort study in the US to systematically evaluate the dual role of multiple comorbidities—as both effect modifiers and potential mediators—in the relationship between long-term PM_2.5_ exposure and incident AD.*

***Comment 18: Three major problems with your study:***

***Comment 18.1: 5y PM_2.5_ is not enough when you are evaluating AD, that takes decades to evolve and progress***

Response: Yes, as noted in our prior response, we acknowledge that our exposure period does not capture exposures earlier in life, prior to Medicare enrollment. As mentioned, we have no data available for earlier exposures, and this limitation has been noted in the Discussion. Nonetheless, we observed a significant association between 5-year average PM_2.5_ exposure prior to disease onset and the incidence of AD and dementia in the Medicare data. It is also likely that ZIP code–specific PM_2.5_ exposure levels during this 5-year period prior to disease onset are correlated with earlier exposures for many study participants.

***Comment 18.2: You have no data on occupational and household exposure and certainly on previous residency expoures, proximity to high traffic, use of public transportation,etc,.***

Response: Yes, as noted above, we do not have personal exposure data for study participants, but only ZIP code–level estimates of ambient exposure. However, data from U.S. studies indicate a reasonably high correlation between personal and ambient PM_2.5_ exposure [23]. We also note that any resulting measurement error is unlikely to be differential (i.e., dependent on disease status) and would therefore likely bias our findings toward the null. Moreover, a substantial body of published literature has now linked relatively recent (in terms of lifetime) ambient PM_2.5_ exposure to AD and dementia, despite the limitation of lacking lifetime exposure data [4, 26].

***Comment 18.3: In Medicare populations the diagnosis of AD is late and once is established as clinicians we know of the overlap and thus, AD diagnosis is seldome crystal clear. Moreover, your clean period is meaningless in the common clinica practice. The patient that was seen because osteoporosis, was able to give her name and address, but nobody ran a cognitive test, so the lack of Dx only means nobody check the cognitive status ( we that see every day, across the US).***

Response: Again, please see our response to the previous comment on this point. Our clean period ensures only the absence of recently recorded AD based on Medicare claims, but it cannot account for a potentially long subclinical or undiagnosed phase of the disease. We have added text to clarify this limitation. Nevertheless, we believe that the research question we address remains important—namely, whether comorbidities mediate the association between PM_2.5_ exposure and AD within the constraints of the available data.

*However, our analysis revealed that the proportion of the association between PM_2.5_ and incident AD mediated by these comorbidities was quite small, with mediated proportions of 1.6% for hypertension, 2.1% for depression, and 4.2% for stroke.*

***Comment 19: This is indeed the most important result of your study. You have to comment about the papers that address the much earlier development of AD in young populations without any evidence of comorbidities. The key for these populations is their exposures from in utero to high PM2.5 concentrations. Biological AD starts in infancy and progresses as the subject is exposed to a complex mixture of pollutants and occupational environments. Our problem as MDs is that we are diagnosing AD late, very late.***

Response: Yes, as noted in response to previous comments, we agree that AD likely begins decades before its clinical diagnosis, with neuropathological processes starting early in life. We have added text in the Discussion to acknowledge this limitation. Our study, however, focuses on the late-life stage of disease development, using national Medicare data to quantify how chronic air pollution exposure and comorbidities during older adulthood contribute to clinically diagnosed AD. The relatively small mediated proportions observed in our analysis suggest that, in this population, air pollution may act primarily through direct neurotoxic and vascular mechanisms rather than indirectly through chronic conditions.

*This study has several key strengths. First, it is the first large-scale national cohort study to simultaneously evaluate the roles of hypertension, stroke, and depression in the association between air pollution and incident AD among US Medicare enrollees.*

***Comment 20: Pollution data is too short.***

Response: Yes, we have responded to this issue above.

*Second, we focused on post-exposure incident cases of hypertension, stroke, and depression as potential mediators, rather than their baseline prevalence, to capture the temporal sequence of exposure, mediators, and AD.*

***Comment 21: “ensuring that conditions such as hypertension, stroke, and depression developed after PM_2.5_ exposure and before the onset of AD.” That is your other problem. If I have a patient with HBP, I do treat him and fully expect neither vascular dementia nor a stroke affects the patient. Your expectation of hypertension, stroke, and depression developed after PM_2.5_ exposure and before the onset of AD. is absolutely not realistic. These people have had PM_2.5_ exposures, but you do not have the data. On the other hand, people are walking with high TAs and they do not know it.***

Response: Our intention was not to imply that hypertension, stroke, or depression arise de novo only after PM_2.5_ exposure, but rather to ensure the correct temporal ordering among exposure, mediator, and outcome within the analytic framework of mediation analysis based on Medicare data. We fully agree that many of these conditions may exist subclinically or remain unrecognized before formal diagnosis, and we have noted this point in the revised manuscript.

**Revised text in the section of Materials and methods:**

**Page 12, Lines 239–245:** We assumed a temporal causal pathway of PM_2.5_→comorbidity→AD. To minimize the potential for reverse causation (AD→comorbidity), comorbidities were required to occur sometime prior to AD diagnosis. Comorbidities, if present, were required to develop between the first year of entry and the occurrence of AD, to ensure they were potential mediators. This temporal structure was designed to ensure proper temporal ordering rather than to imply short-term causal effects. In practice, these comorbidities were first diagnosed in our mediation analysis several years before the first recorded AD diagnosis in the Medicare data.

*Despite these strengths, our study has several limitations. First, while the exposure prediction model performs well, there is a potential measurement error because PM_2.5_ levels were assigned based on ZIP code instead of individual residential addresses. However, other recent work has suggested that measurement error in our modeled exposure data would not have a strong effect* [27, 28]*. Third, although we accounted for multiple covariates, these data were only available at the ZIP code level. Consequently, residual or unmeasured individual-level confounders, such as alcohol consumption, smoking history, and physical activity, may bias the observed findings.*

*In conclusion, long-term PM_2.5_ exposure was associated with increased AD risk in a large national cohort, primarily through direct pathways rather than mediation by common comorbidities.*

***Comment 22: This is your absolute beautiful finding, take advantage and expand the results using available literature, you will help to change the current paradigm where you need comorbidities to develop AD. WRONG!***

Response: We thank the reviewer for this encouraging comment.

**Reviewer #3:**

***Comment 1: The authors report a very interesting large study using administrative data from Medicare beneficiaries in the United States to investigate the association between exposure to air pollution and incident Alzheimer's disease. While other studies have done this, the authors evaluated whether the association was mediated by hypertension, stroke and depression. The latter three risk factors for dementia are also impacted by exposure to air pollution. This study therefore attempts to shed light on the direct vs indirect effect of air pollution on dementia which is important for elucidating potential mechanisms and also interventions. The paper has several strengths. The sample size is huge and the study period is 18 years. The research question is important because of the large number of people exposed to air pollution and the high prevalence of the three risk factors.***
Response: We thank the reviewer for acknowledging the importance of our work. Detailed responses to subsequent comments are provided below.

***There are some limitations of the study which reduce the clarify of findings their potential significance.***
***Comment 2: The authors mention adjusting for covariates including measures of socioeconomic status such as percentage of population below the poverty line, percentage with less than high school education as these covariates have been associated with AD and PM. It appears that these covariates were not adjusted for individuals, but for locations. This is a limitation in the methodology - presumably due to lack of demographic data in the Medicare data. Sociodemographic variables are the biggest confounders of the association between the key variables ie people with low education and low SES are more likely to develop chronic diseases, and AD so the way these are handled in the analysis is very important for interpretation of results. Further information needs to be provided on the methods used to adjust for these variables and the results for the associations between these confounders and the exposure and outcome should be reported. This will assist in interpreting the findings.***

Response: We thank the reviewer for this comment. We agree that socioeconomic status (SES) and related demographic characteristics are major determinants of both long-term PM₂.₅ exposure and Alzheimer’s disease (AD) risk. Individual-level education and income data are unavailable in Medicare records; therefore, we used validated multilevel proxies to capture socioeconomic context. Specifically, we adjusted for individual-level demographic variables including age, sex, race, and Medicaid dual eligibility. Although Medicaid dual eligibility does not directly measure education or employment, it is a validated proxy for low income and financial hardship and is commonly used to capture individual-level socioeconomic disadvantage in large administrative datasets [29]. Our primary models also adjusted for incorporated time-varying ZIP code– and county-level measures, including the percentage of population below the poverty line, percentage with less than a high school education, median household income, population density, and county-level behavioral indicators (smoking rate and mean BMI). Validation studies have shown that area-based socioeconomic indicators yield results consistent in direction and only slightly attenuated compared with those using individual-level SES data, suggesting that any residual misclassification would likely bias our results slightly toward the null [1-3]. These covariates were updated annually to reflect changes in local socioeconomic environments. These findings indicate that while individual-level SES data are lacking, area-level proxies capture substantial socioeconomic variability relevant to both exposure and outcome, and residual confounding is unlikely to fully account for the observed associations.

Regarding the methods used to adjust for these variables, covariate selection was informed by a causal framework and prior literature linking PM_2.5_ exposure, socioeconomic conditions, comorbidities, and AD to ensure appropriate adjustment for confounding while avoiding overadjustment [4, 6, 7]. This approach is consistent with best practices in national Medicare-based epidemiologic research and allows integration of both individual- and contextual-level determinants. We have clarified these methodological details in the revised Methods section and added explanatory text in the Discussion to emphasize how socioeconomic factors were addressed and their potential influence on interpretation of results. In addition, as requested here, we have included the full model results for the associations between these confounders and outcome in the Supplementary Materials of the revised manuscript (see S2 Table).

**Revised text in the section of Materials and Methods:**

**Page 10, Lines 193–198:** We adjusted for several covariates, including study year and individual-level characteristics (age at entry, year of entry, sex, race, and Medicaid eligibility), as well as a number of ZIP code-level average SES indicators. Although Medicaid dual eligibility does not directly measure education or employment, it serves as a validated proxy for low income and financial hardship and is widely used as an indicator of individual-level socioeconomic disadvantage in Medicare-based studies [29].

**Revised text in the section of Materials and Methods:**

**Page 11, Lines 205–208:** These covariates were selected based on prior literature indicating that they could be associated with PM_2.5_ exposure, comorbidities, and AD, and therefore may confound the observed relationships between PM_2.5_ and AD, as well as between PM_2.5_ and comorbidities [4, 6, 7].

**Revised text in the section of Results:**

**Page 16, Lines 327–331:** In the overall population, a higher hazard ratio (HR) for AD was observed with each IQR increase in the 5-year average concentrations of PM_2.5_, with an HR of 1.085 (95% CI: 1.078, 1.091), after adjusting for all covariates and three comorbidities (full model results can be found in S2 Table).

**Revised text in the section of Discussion:**

**Page 21, Lines 452–465:** Third, although we accounted for multiple covariates, some data were only available at the ZIP code level. Consequently, we were able to adjust for some risk factors, such as smoking and BMI, only at the area level, which may have introduced some residual confounding in the PM_2.5_–AD or PM_2.5_–comorbidity associations, particularly if these unmeasured individual behaviors were correlated with air pollution levels. However, we adjusted for individual-level Medicaid eligibility and race, both important indicators of SES, as well as other key individual-level confounders, i.e., age and sex. Moreover, validation literature has shown that area-based socioeconomic indicators yield results consistent in direction and slightly attenuated compared with those using individual-level SES data, suggesting that any residual misclassification would likely bias our results only slightly toward the null [1-3]. Overall, we believe that our control for a large number of individual- and area-level potential confounders was reasonably thorough, and that confounding was unlikely to markedly distort our findings for either the PM_2.5_/AD associations or the PM_2.5_/mediator associations.

***Comment 3: Was there any demographic information in the Medicare database eg. on education etc. that could have provided individual level SES variables?***

Response: The Medicare claims data used in our study do not contain individual-level sociodemographic information such as education, income, or occupation. However, we included Medicaid dual eligibility as an individual-level indicator of low income and financial hardship, which has been widely validated as a socioeconomic proxy in Medicare-based epidemiologic research [29]. In addition, ZIP code– and county-level socioeconomic variables—including poverty rate, educational attainment, and median household income—were incorporated from the U.S. Census and American Community Survey to represent contextual SES. These proxies have been shown in prior large-scale Medicare studies to effectively reduce residual confounding by socioeconomic status [4, 6, 29, 30]. Moreover, recent causal inference analyses using the same Medicare data and generalized propensity score (GPS) methods found that even after balancing numerous area-level socioeconomic and health-related variables, the associations between PM_2.5_ exposure and Alzheimer disease and related dementias remained robust and directionally consistent with traditional regression models [31]. Together, these approaches provide a reasonable and validated strategy for addressing socioeconomic confounding in the absence of direct individual-level SES data. We have added the related content in the revised manuscript.

**Revised text in the section of Materials and Methods:**

**Page 10, Lines 193–198:** We adjusted for several covariates, including study year and individual-level characteristics (age at entry, year of entry, sex, race, and Medicaid eligibility), as well as a number of ZIP code-level average SES indicators. Although Medicaid dual eligibility does not directly measure education or employment, it serves as a validated proxy for low income and financial hardship and is widely used as an indicator of individual-level socioeconomic disadvantage in Medicare-based studies [29].

**Revised text in the section of Discussion:**

**Page 21, Lines 452–465:** Third, although we accounted for multiple covariates, some data were only available at the ZIP code level. Consequently, we were able to adjust for some risk factors, such as smoking and BMI, only at the area level, which may have introduced some residual confounding in the PM_2.5_–AD or PM_2.5_–comorbidity associations, particularly if these unmeasured individual behaviors were correlated with air pollution levels. However, we adjusted for individual-level Medicaid eligibility and race, both important indicators of SES, as well as other key individual-level confounders, i.e., age and sex. Moreover, validation literature has shown that area-based socioeconomic indicators yield results consistent in direction and slightly attenuated compared with those using individual-level SES data, suggesting that any residual misclassification would likely bias our results only slightly toward the null [1-3]. Overall, we believe that our control for a large number of individual- and area-level potential confounders was reasonably thorough, and that confounding was unlikely to markedly distort our findings for either the PM_2.5_/AD associations or the PM_2.5_/mediator associations.

***Comment 4: Is there way of evaluating aggregation bias caused by using the population level measures of SES? What degree of misclassification can occur using this methodology? I think this needs to be explained far better to assure readers that the methodology for this adjustment is adequate and the associations reported are reliable.***

Response: We acknowledge the potential for aggregation (ecological) bias when population-level socioeconomic indicators are used as proxies for individual-level SES. Nevertheless, prior validation studies have shown that well-chosen area-based SES measures capture population-level variation in SES and yield estimates of health inequalities consistent in direction and slightly attenuated compared with those based on individual SES. In particular, Subramanian et al. (2006) [1] found that well-chosen area-based SES indicators yield estimates of health inequalities comparable in direction and magnitude to those based on individual SES, albeit slightly attenuated, and thus serve as reliable contextual surrogates. Similarly, Moss et al. (2021) [2] reported moderate concordance between SES metrics at the individual, census-tract, and county levels and found that using area-level proxies systematically underestimates, rather than exaggerates, SES–health associations. These findings suggest that any misclassification from our use of ZIP- and county-level SES indicators would likely bias our estimates toward the null, rendering our results conservative rather than inflated.

In our study, we believe we have mitigated aggregation bias (residual confounding due to the use of areal-level covariates, e.g., for SES) by combining both individual-level SES variables (Medicaid dual eligibility and, to some extent, race) and area-level indicators (poverty, education, and income), which were updated annually to reflect changing socioeconomic contexts. We believe that any aggregation-related bias is unlikely to account for the observed associations and, if present, would bias our estimates toward the null rather than away from it. We have clarified this rationale in the revised manuscript.

**Revised text in the section of Materials and Methods:**

**Page 10, Lines 193–198:** We adjusted for several covariates, including study year and individual-level characteristics (age at entry, year of entry, sex, race, and Medicaid eligibility), as well as a number of ZIP code-level average SES indicators. Although Medicaid dual eligibility does not directly measure education or employment, it serves as a validated proxy for low income and financial hardship and is widely used as an indicator of individual-level socioeconomic disadvantage in Medicare-based studies [29].

**Revised text in the section of Discussion:**

**Page 21, Lines 452–465:** Third, although we accounted for multiple covariates, some data were only available at the ZIP code level. Consequently, we were able to adjust for some risk factors, such as smoking and BMI, only at the area level, which may have introduced some residual confounding in the PM_2.5_–AD or PM_2.5_–comorbidity associations, particularly if these unmeasured individual behaviors were correlated with air pollution levels. However, we adjusted for individual-level Medicaid eligibility and race, both important indicators of SES, as well as other key individual-level confounders, i.e., age and sex. Moreover, validation literature has shown that area-based socioeconomic indicators yield results consistent in direction and slightly attenuated compared with those using individual-level SES data, suggesting that any residual misclassification would likely bias our results only slightly toward the null [1-3]. Overall, we believe that our control for a large number of individual- and area-level potential confounders was reasonably thorough, and that confounding was unlikely to markedly distort our findings for either the PM_2.5_/AD associations or the PM_2.5_/mediator associations.

**References**

1. Subramanian SV, Chen JT, Rehkopf DH, Waterman PD, Krieger N. Comparing individual- and area-based socioeconomic measures for the surveillance of health disparities: A multilevel analysis of Massachusetts births, 1989-1991. Am J Epidemiol. 2006; 164:823-834. http://doi.org/10.1093/aje/kwj313 PMID: 16968866

2. Moss JL, Johnson NJ, Yu M, Altekruse SF, Cronin KA. Comparisons of individual- and area-level socioeconomic status as proxies for individual-level measures: Evidence from the mortality disparities in American communities study. Popul Health Metr. 2021; 19:1. http://doi.org/10.1186/s12963-020-00244-x PMID: 33413469

3. Link-Gelles R, Westreich D, Aiello AE, Shang N, Weber DJ, Holtzman C, et al. Bias with respect to socioeconomic status: A closer look at zip code matching in a pneumococcal vaccine effectiveness study. SSM Popul Health. 2016; 2:587-594. http://doi.org/10.1016/j.ssmph.2016.08.005 PMID: 27668279

4. Shi L, Steenland K, Li H, Liu P, Zhang Y, Lyles RH, et al. A national cohort study (2000-2018) of long-term air pollution exposure and incident dementia in older adults in the United States. Nat Commun. 2021; 12:6754. http://doi.org/10.1038/s41467-021-27049-2 PMID: 34799599

5. Qiu X, Shi L, Kubzansky LD, Wei Y, Castro E, Li H, et al. Association of long-term exposure to air pollution with late-life depression in older adults in the US. JAMA Netw Open. 2023; 6:e2253668. http://doi.org/10.1001/jamanetworkopen.2022.53668 PMID: 36763364

6. Shi L, Zhu Q, Wang Y, Hao H, Zhang H, Schwartz J, et al. Incident dementia and long-term exposure to constituents of fine particle air pollution: A national cohort study in the United States. Proc Natl Acad Sci U S A. 2023; 120:e2211282119. http://doi.org/10.1073/pnas.2211282119 PMID: 36574646

7. Steenland K, Tan Y, Wingo T, Shi L, Xiao S, Wharton W. The effect of race and co-morbidities on Alzheimer's Disease based on Medicare data. Alzheimers Dement. 2023; 19:1858-1864. http://doi.org/10.1002/alz.12838 PMID: 36327171

8. Grande G, Ljungman P, Eneroth K, Bellander T, Rizzuto D. Association between cardiovascular disease and long-term exposure to air pollution with the risk of dementia. JAMA Neurol. 2020; 77:801-809. http://doi.org/10.1001/jamaneurol.2019.4914 PMID: 32227140

9. Ilango SD, Chen H, Hystad P, van Donkelaar A, Kwong JC, Tu K, et al. The role of cardiovascular disease in the relationship between air pollution and incident dementia: A population-based cohort study. Int J Epidemiol. 2020; 49:36-44. http://doi.org/10.1093/ije/dyz154 PMID: 31347651

10.Zhang B, Langa KM, Weuve J, D'Souza J, Szpiro A, Faul J, et al. Hypertension and stroke as mediators of air pollution exposure and incident dementia. JAMA Netw Open. 2023; 6:e2333470. http://doi.org/10.1001/jamanetworkopen.2023.33470 PMID: 37728927

11. Vanderweele TJ. Mediation analysis: A practitioner's guide. Annu Rev Public Health. 2016; 37:17-32. http://doi.org/10.1146/annurev-publhealth-032315-021402 PMID: 26653405

12. Willroth EC, Pfund GN, Rule PD, Hill PL, John A, Kyle K, et al. A review of the literature on wellbeing and modifiable dementia risk factors. Ageing Res Rev. 2024; 99:102380. http://doi.org/10.1016/j.arr.2024.102380 PMID: 38880341

13. Cristaldi A, Fiore M, Oliveri CG, Pulvirenti E, Favara C, Grasso A, et al. Possible association between PM(2.5) and neurodegenerative diseases: A systematic review. Environ Res. 2022; 208:112581. http://doi.org/10.1016/j.envres.2021.112581 PMID: 34979121

14. Peters A. Ambient Air Pollution and Alzheimer's Disease: The Role of the composition of fine particles. Proc Natl Acad Sci U S A. 2023; 120:e2220028120. http://doi.org/10.1073/pnas.2220028120 PMID: 36626559

15. Calderon-Garciduenas L, Ayala A, Mukherjee PS. 2024 United States elections: Air pollution, neurodegeneration, neuropsychiatric, and neurodevelopmental disorders. Who cares? J Alzheimers Dis. 2024; 98:1277-1282. http://doi.org/10.3233/JAD-231373 PMID: 38517792

16. Calderon-Garciduenas L, Cejudo-Ruiz FR, Gonzalez-Maciel A, Reynoso-Robles R, Ramos-Morales A, Perez-Guille BE, et al. Cardiovascular damage, arrhythmogenesis and overlapping Alzheimer and Parkinson's Diseases and TDP-43 pathology start in pediatric and young adult urban populations: Magnetic ultrafine particulate matter and industrial particles with motion behavior play a key role. Environ Res. 2025; 285:122558. http://doi.org/10.1016/j.envres.2025.122558 PMID: 40803397

17. Calderon-Garciduenas L, Gonzalez-Maciel A, Reynoso-Robles R, Delgado-Chavez R, Mukherjee PS, Kulesza RJ, et al. Hallmarks of Alzheimer Disease are evolving relentlessly in metropolitan Mexico city infants, children and young adults. APOE4 carriers have higher suicide risk and higher odds of reaching NFT stage V at </= 40 years of age. Environ Res. 2018; 164:475-487. http://doi.org/10.1016/j.envres.2018.03.023 PMID: 29587223

18. Karanth SD, Schmitt FA, Nelson PT, Katsumata Y, Kryscio RJ, Fardo DW, et al. Four common late-life cognitive trajectories patterns associate with replicable underlying neuropathologies. J Alzheimers Dis. 2021; 82:647-659. http://doi.org/10.3233/JAD-210293 PMID: 34057090

19. Kang YJ, Tan HY, Lee CY, Cho H. An air particulate pollutant induces neuroinflammation and neurodegeneration in human brain models. Adv Sci (Weinh). 2021; 8:e2101251. http://doi.org/10.1002/advs.202101251 PMID: 34561961

20. Hahad O, Lelieveld J, Birklein F, Lieb K, Daiber A, Munzel T. Ambient air pollution increases the risk of cerebrovascular and neuropsychiatric disorders through induction of inflammation and oxidative stress. Int J Mol Sci. 2020; 21. http://doi.org/10.3390/ijms21124306 PMID: 32560306

21. Kilian J, Kitazawa M. The emerging risk of exposure to air pollution on cognitive decline and Alzheimer's Disease - evidence from epidemiological and animal studies. Biomed J. 2018; 41:141-162. http://doi.org/10.1016/j.bj.2018.06.001 PMID: 30080655

22. Jin T, Di Q, Requia WJ, Danesh Yazdi M, Castro E, Ma T, et al. Associations between long-term air pollution exposure and the incidence of cardiovascular diseases among American older adults. Environ Int. 2022; 170:107594. http://doi.org/10.1016/j.envint.2022.107594 PMID: 36283157

23. Boomhower SR, Long CM, Li W, Manidis TD, Bhatia A, Goodman JE. A review and analysis of personal and ambient PM(2.5) measurements: Implications for epidemiology studies. Environ Res. 2022; 204:112019. http://doi.org/10.1016/j.envres.2021.112019 PMID: 34534524

24. Taylor DJ, Ostbye T, Langa KM, Weir D, Plassman BL. The accuracy of Medicare claims as an epidemiological tool: The case of dementia revisited. J Alzheimers Dis. 2009; 17:807-815. http://doi.org/10.3233/JAD-2009-1099 PMID: 19542620

25. Alzheimer's Association 2024 Alzheimer's Disease facts and figures. Alzheimers Dement. 2024 May;20(5):3708-3821. http://doi.org/10.1002/alz.13809 PMID: 38689398

26. Wilker EH, Osman M, Weisskopf MG. Ambient air pollution and clinical dementia: Systematic review and meta-analysis. BMJ. 2023; 381:e071620. http://doi.org/10.1136/bmj-2022-071620 PMID: 37019461

27. Wei Y, Qiu X, Yazdi MD, Shtein A, Shi L, Yang J, et al. The impact of exposure measurement error on the estimated concentration-response relationship between long-term exposure to PM_2.5_ and mortality. Environ Health Perspect. 2022; 130:77006. http://doi.org/10.1289/EHP10389 PMID: 35904519

28. Feng Y, Wei Y, Coull BA, Schwartz JD. Measurement error correction for ambient PM(2.5) exposure using stratified regression calibration: Effects on all-cause mortality. Environ Res. 2023; 216:114792. http://doi.org/10.1016/j.envres.2022.114792 PMID: 36375508

29. Josey KP, Delaney SW, Wu X, Nethery RC, Desouza P, Braun D, et al. Air pollution and mortality at the intersection of race and social class. N Engl J Med. 2023; 388:1396-1404. http://doi.org/10.1056/NEJMsa2300523 PMID: 36961127

30. Di Q, Wang Y, Zanobetti A, Wang Y, Koutrakis P, Choirat C, et al. Air pollution and mortality in the Medicare population. N Engl J Med. 2017; 376:2513-2522. http://doi.org/10.1056/NEJMoa1702747 PMID: 28657878

31. Qin MM, Khoshnevis N, Dominici F, Braun D, Zanobetti A, Mork D. Comparing traditional and causal inference methodologies for evaluating impacts of long-term air pollution exposure on hospitalization with Alzheimer Disease and related dementias. Am J Epidemiol. 2025; 194:64-72. http://doi.org/10.1093/aje/kwae133 PMID: 38907309
