# Supplementary material for: The role of comorbidities in the associations between air pollution and Alzheimer’s disease: A national cohort study in the American Medicare population
Source: PLoS Med. 2026 Feb 17;23(2):e1004912. doi: 10.1371/journal.pmed.1004912 (PMC12912588; doi:10.1371/journal.pmed.1004912)
Supplement: Attachment — Submitted filename: Response_to_reviewers_auresp_3.docx [file pmed.1004912.s009.docx]

**Response Letter to the Reviewers’ Comments**

**Manuscript reference number: PMEDICINE-D-25-02362R2**

**Title: The Role of Comorbidities in the Associations between Air Pollution and Alzheimer's Disease: A National Cohort Study in the American Medicare Population**

The Authors thank the Reviewers for the feedback that they provided and the Editor for the opportunity to respond to these comments. Detailed responses to each comment are given below. Reviewer/Editor comments are in **bold** *italics*. The author’s responses are in plain text. Modifications to the manuscript are in red. Page and line numbers in the responses correspond to changes in the revised manuscript (highlighted in blue).

******************************************************************************

**Comments from the Editor:**

***I have discussed the paper with my colleagues and the academic editor and it was also seen again by 2 reviewers. I am pleased to say that provided the remaining editorial and production issues are dealt with we are planning to accept the paper for publication in the journal after some minor concerns from the academic editor and the reviewers are addressed. Specifically, the academic editor asked for some further discussion of the potential impact that AD misdiagnosis may have on the results, especially given that the increase in the risk for AD is rather small. Moreover, he raised concerns about the contribution of vascular mechanisms to the increased risk for AD in participants who suffered from stroke(s). One of the reviewers asked for for a further justification of the 5-year duration of the clean period. The remaining issues that need to be addressed are listed in more detail at the end of this email. Any accompanying reviewer attachments can be seen via the link below.***

Response: We appreciate the opportunity to address the remaining minor concerns prior to acceptance. We have carefully considered all comments from the Editor, the academic editor, and the reviewers, and have addressed them in the revised manuscript. Detailed responses are provided below.

**Comments from the Academic Editor:**

***The Academic Editor expressed concerns about how the clinical diagnosis was conducted and how it may have impacted the results: Authors used the ICD-9/10 codes; however, a paper from Pippenger et al., 2001 that looked at "Neurologists' use of ICD-9 codes for dementia" concluded that "The specific code for AD, 331.0, was used for only 36.5% of patients judged by the neurologist to have AD as the most likely diagnosis. Other codes used were not inaccurate but would result in lower reimbursement". With this in mind and given the small increase in the risk for AD, this inaccuracy in diagnosis could be quite meaningful.*** ***Moreover, even when AD is a reasonable diagnosis, there is comorbid vascular or other pathology, which may make the difference to whether the patient presents with dementia or not. My particular query is about vascular pathology. The fact that stroke increased the risk makes me suspect that vascular mechanisms are possibly making a contribution. There is much evidence to suggest that cerebrovascular disease and AD are independent processes that synergistically contribute to cognitive impairment.***

Response: We fully acknowledge the limitations of using ICD-9/10 codes to identify AD in administrative claims data. As noted by Pippenger et al. (2001), AD-specific codes may be underused in clinical practice, in part due to reimbursement considerations, and clinicians may initially code dementia more broadly before specifying AD. Importantly, this limitation is well recognized in Medicare-based research and has motivated the development of validated Chronic Conditions Warehouse (CCW) algorithms that incorporate multiple claim sources and longitudinal information. Prior validation studies have shown that AD diagnoses in Medicare claims have high specificity (approximately 95%) and moderate sensitivity (approximately 64%), indicating that while some true cases may be missed, false positives are relatively uncommon [1, 2]. To address this concern of outcome misclassification, we used the validated Medicare Chronic Conditions Warehouse (CCW) definition for AD and implemented complementary strategies in prior Medicare-based work to evaluate the potential impact of outcome misclassification (as described in the Supplemental material of the referenced Nature Communications study) [3]. First, we fit a linear rate model (events/person-time) using generalized estimating equation, which provides an additive effect estimate and is less sensitive to random outcome misclassification, because random misclassification tends to be absorbed into the residual error rather than substantially biasing coefficients. Second, we implemented a quantitative bias-correction approach in which we adjusted ZIP-level AD case counts using published Medicare claims sensitivity (0.64) and specificity (0.95) estimated derived from clinically adjudicated diagnoses [1] and re-estimated exposure coefficients within a simplified Poisson framework, then mapped the correction back to the Cox model estimates. Across both approaches, the corrected estimates were very similar to the primary results, indicating that plausible levels of AD outcome misclassification would bias associations slightly toward the null rather than create spurious positive findings [3]. We have added related content to the Discussion in our original manuscript (see **Pages 20-21**, **Lines 452-462**).

Additionally, we fully agree that AD frequently co-occurs with cerebrovascular pathology and that neurodegenerative and vascular processes often act synergistically rather than independently in the development of cognitive impairment. Our study design and analytic framework explicitly account for this complexity. Rather than assuming mutually exclusive disease entities, we examined stroke as both a potential mediator and an effect modifier in the association between PM_2.5_ exposure and incident AD. Notably, we observed modest effect modification by stroke, with stronger PM_2.5_–AD associations among individuals with a history of stroke. This finding is biologically plausible and aligns with existing evidence suggesting that cerebrovascular injury may increase vulnerability to environmental neurotoxicants by impairing blood–brain barrier integrity, cerebral perfusion, and neurovascular coupling. At the same time, our formal mediation analyses showed that only a small proportion of the PM_2.5_–AD association was mediated through incident stroke, indicating that vascular pathology alone does not fully explain the observed relationship. These findings support an interpretation in which cerebrovascular disease may act as a susceptibility factor that amplifies these effects rather than serving as the primary causal pathway. We have clarified these points in the Discussion in our revised manuscript.

**Revised text in the section of Discussion:**

**Page 19, Lines 417–425:** Additionally, individuals with stroke may experience altered cerebral perfusion or impaired glymphatic clearance, both of which may interact synergistically with environmental insults to accelerate neurodegeneration [4]. These findings align with emerging mechanistic studies that highlight shared pathways between cerebrovascular injury and pollution-induced neural damage [5-7]. The stronger PM_2.5_–AD association observed among individuals with stroke supports a synergistic model in which cerebrovascular pathology increases vulnerability to pollution-related neurodegenerative processes, rather than indicating that vascular disease alone drives the observed association.

**Comments from the Reviewers:**

**Reviewer #1:** ***We thank the authors for addressing our previous concerns. The 5-year duration of the clean period before cohort entry (in the reply to Comment 5) might be further justified, if possible.***

Response: We implemented a 5-year clean period after Medicare enrollment, during which no AD–related diagnosis codes were recorded, to reduce the likelihood of misclassifying prevalent but previously undocumented cases as incident outcomes. Given the long preclinical phase of AD and the delay between symptom onset and diagnosis in administrative claims, a multi-year clean period improves the specificity of incident case identification in Medicare data. While there is no definitive answer to the question of optimal clean periods, we selected a 5-year window as a pragmatic balance between more stringent exclusion of prevalent disease and preservation of cohort size and generalizability, particularly given the long latency of AD, and to maintain consistency with prior Medicare-based studies that examined AD or dementia as an outcome [3, 8, 9]. We therefore consider a 5-year clean period to be a reasonable and widely accepted approach for approximating incident AD in Medicare claims data. We have clarified this rationale and cited relevant precedent in the revised manuscript.

**Revised text in the section of Materials and Methods:**

**Page 8, Lines 145–152:** To better capture newly recorded AD diagnoses rather than previously documented cases, we implemented a 5-year “clean period” prior to cohort entry. This approach aims to minimize misclassification of prevalent cases as incident based on Medicare claims, but does not imply that participants were biologically free of early disease during this period. Given the long preclinical phase of AD and the delay between symptom onset and diagnosis in administrative data, we considered a 5-year period to be a reasonable and widely used approach for reducing the likelihood of including previously diagnosed AD cases in Medicare-based studies of AD or dementia [3, 8, 9].

**References**

1. Taylor DJ, Ostbye T, Langa KM, Weir D, Plassman BL. The accuracy of Medicare claims as an epidemiological tool: The case of dementia revisited. J Alzheimers Dis. 2009; 17:807-815. http://doi.org/10.3233/JAD-2009-1099 PMID: 19542620

2. Taylor DJ, Fillenbaum GG, Ezell ME. The accuracy of Medicare claims data in identifying Alzheimer's disease. J Clin Epidemiol. 2002; 55:929-937. http://doi.org/10.1016/s0895-4356(02)00452-3 PMID: 12393082

3. Shi L, Steenland K, Li H, Liu P, Zhang Y, Lyles RH, et al. A national cohort study (2000-2018) of long-term air pollution exposure and incident dementia in older adults in the United States. Nat Commun. 2021; 12:6754. http://doi.org/10.1038/s41467-021-27049-2 PMID: 34799599

4. Toth P, Tarantini S, Csiszar A, Ungvari Z. Functional vascular contributions to cognitive impairment and dementia: Mechanisms and consequences of cerebral autoregulatory dysfunction, endothelial impairment, and neurovascular uncoupling in aging. Am J Physiol Heart Circ Physiol. 2017; 312:H1-H20. http://doi.org/10.1152/ajpheart.00581.2016 PMID: 27793855

5. Mariani E, Polidori MC, Cherubini A, Mecocci P. Oxidative stress in brain aging, neurodegenerative and vascular diseases: An overview. J Chromatogr B Analyt Technol Biomed Life Sci. 2005; 827:65-75. http://doi.org/10.1016/j.jchromb.2005.04.023 PMID: 16183338

6. Liu XQ, Huang J, Song C, Zhang TL, Liu YP, Yu L. Neurodevelopmental toxicity induced by PM_2.5_ Exposure and its possible role in neurodegenerative and mental disorders. Hum Exp Toxicol. 2023; 42:9603271231191436. http://doi.org/10.1177/09603271231191436 PMID: 37537902

7. Steven S, Frenis K, Oelze M, Kalinovic S, Kuntic M, Bayo JM, et al. Vascular inflammation and oxidative stress: Major triggers for cardiovascular disease. Oxid Med Cell Longev. 2019; 2019:7092151. http://doi.org/10.1155/2019/7092151 PMID: 31341533

8. Zhang H, Wang Y, Li H, Zhu Q, Ma T, Liu Y, et al. The role of the components of PM(2.5) in the incidence of Alzheimer's disease and related disorders. Environment international. 2025; 200:109539. http://doi.org/10.1016/j.envint.2025.109539 PMID:

9. Steenland K, Tan Y, Wingo T, Shi L, Xiao S, Wharton W. The effect of race and co-morbidities on Alzheimer's disease based on Medicare data. Alzheimers Dement. 2023; 19:1858-1864. http://doi.org/10.1002/alz.12838 PMID: 36327171
